# Combining tethered and untethered magnetic robots via a magnetically triggerable latch for target payload delivery and retrieval

**DOI:** 10.1126/sciadv.adu6025

**Published:** 2026-01-01

**Authors:** Michael Brockdorff, Benjamin Calmé, Tianlu Wang, Luke J. Tinsley, Joshua Davy, Peter Lloyd, James H. Chandler, Russell A. Harris, Metin Sitti, Pietro Valdastri

**Affiliations:** ^1^ STORM Lab, Institute of Autonomous Systems and Sensing (IRASS), School of Electronic and Electrical Engineering, University of Leeds, Leeds, UK.; ^2^ Physical Intelligence Department, Max Planck Institute for Intelligent Systems, 70569 Stuttgart, Germany.; ^3^ Department of Mechanical Engineering, University of Hawaiʻi at Mānoa, Honolulu, HI 96822, USA.; ^4^ School of Mechanical Engineering, University of Leeds, Leeds, UK.; ^5^ Department of Engineering, Manchester Metropolitan University, Manchester, UK.; ^6^ School of Medicine and College of Engineering, Koç University, 34450 Istanbul, Turkey.

## Abstract

The reach and scope of minimally invasive surgical procedures can be transformed via the development of continuum robots. Through soft, flexible structures and accurate navigation, previously inaccessible anatomical regions can be safely reached. Dependent on both actuation mode and clinical application, however, rigidity and miniaturization potential can still present substantial challenges. Magnetic soft continuum robots (mSCRs) offer promising solutions to these key questions. Furthermore, micrometer- to millimeter-scale untethered magnetic robots (mUMRs) offer unparalleled miniaturization potential enabling targeted therapeutic delivery. Leveraging the benefits of magnetic actuation, this study introduces a bespoke, continuously magnetized catheter that synergizes the navigational strengths of mSCRs with the functional effectiveness of mUMRs to precisely deliver drug-doped payloads to otherwise unreachable regions deep within the anatomy. In particular, this system uses a magnetic latching mechanism, ensuring precise drug delivery and efficient retrieval, demonstrated in an ex vivo porcine kidney model for organ transplantation–related immunosuppressant delivery.

## Teaser

A magnetic latch enables tethered magnetic soft robots to deliver and retrieve drug-coated, wireless, small-scale magnetic robots.

## INTRODUCTION

Medical procedures have undergone substantial transformations over the past century (*1*), with the advent of slender, maneuverable instruments paving the way for minimally invasive surgery (MIS). MIS offers reduced access trauma and surgical morbidity and decreases the risk of infection (*2*). However, the instruments typically used in MIS are rigid, requiring the nonlinear pathways of the human anatomy to conform to their shape. This can lead to substantial damage to sensitive structures, such as those in the pancreas and kidneys, necessitating open surgery. In contrast, soft and compliant continuum robots (CRs), which theoretically have infinite degrees of freedom (DoFs), offer smooth curvilinear motion. This capability allows navigation through convoluted lumens, enabling, for example, the use of natural anatomical orifices to perform an internal incision as close as possible to the targeted area, thereby minimizing trauma to the patient (*3*).

CRs are usually controlled via an external tether, allowing them to be effectively used in various applications, including cardiac (*4*), neurosurgical (*5*), early breast cancer diagnosis (*6*), and bronchoscopy (*7*). A recent class of CRs that have shown suitability for medical applications is magnetic soft continuum robots (mSCRs). These devices make use of integrated permanent magnets (*8*–*11*) or electromagnetic coils (*12*, *13*) or use hard magnetic particles embedded throughout their body (*14*). Actuation of mSCRs is achieved via the application of external wireless magnetic fields, removing the need for a direct external connection for actuation and facilitating simple miniaturization to the millimeter scale. Although control of mSCRs is nontrivial, control techniques have been demonstrated for tip-driven mSCRs (*9*), mSCRs capable of achieving predefined shapes (*15*) and follow-the-leader full-shape control (*16*). Despite their navigational prowess, making mSCRs suitable as guidewires (*14*), functionalization below the millimeter scale remains elusive. Photothermal delivery (*17*) and mSCRs capable of ultrasound imaging (*18*) have been demonstrated. However, these require additional components inserted along the length of the mSCR, increasing overall diameter and stiffness.

In contrast, untethered medical robots are capable of extreme miniaturization (*19*) and have been functionalized to demonstrate effectiveness in applications such as tissue biopsy (*20*), biome sampling (*21*), drug delivery (*22*), and stem cell transplantation (*23*, *24*), among others (*25*). The majority of these robots use some form of onboard actuation to propel and control them within the body. Similar to their tethered counterparts, remote actuation techniques, such as those based on light (*26*), ultrasound (*27*), and magnetic fields (*28*), eliminate the need for onboard actuation, enabling further size reduction. Unlike light-based control, which requires a clear line of sight, and ultrasound, which can be affected by acoustic impedance mismatch and requires a medium to propagate through, magnetic fields can be imparted, without signal loss, through living tissue. This allows for effective control of untethered robots deep within the body. An example of such a millimeter-scale untethered magnetic robot (mUMR) is the stent-shaped magnetic soft robot (*29*). The soft adaptive structure and high magnetic moment of this device allow it to be navigated and controlled into the small vasculature of the brain where potentially lifesaving drugs may be deployed (*30*).

Despite the versatility and theoretical effectiveness of mUMRs, challenges toward clinical application remain. The drug-doped surface of the mUMR is immediately exposed to the body’s anatomy upon introduction. This could pose complications, particularly when a specific organ is targeted for treatment, as inadvertent drug exposure could result in severe, potentially fatal, toxicity to other organs (*31*). This highlights the need for precise payload delivery in the vicinity of the target organs for the application of these promising technological tools in medical treatments. Second, small volumes and the need for biocompatibility typically result in magnetic milli-robots having a low responsiveness to magnetic fields (*32*). This makes navigation challenging and requires machinery to generate very large magnetic fields, resulting in higher overall cost, reduced workspace, and safety issues. In a drive to increase the magnetic moment, nonbiocompatible magnets can be used, such as permanent magnets or neodymium-iron-boron (NdFeB) microparticles. However, this results in the need for retrieval of the mUMRs once the procedure has been completed.

In the meantime, the field of organ transplantation could substantially benefit from advancements in mUMRs for targeted drug delivery. Organ transplantation represents one of humanity’s greatest challenges, with its first successful procedure, a kidney transplant, performed 70 years ago (*33*). The current state of transplantation continues to rely on systemic immunosuppression methods developed over three decades ago (*34*). Despite a declining incidence of clinical rejection, there are substantial disparities in organ availability and recipient needs. In addition, the morbidity and mortality associated with systemic immunosuppression persist, with no progress in reducing the rate of graft failure due to chronic rejection and a lack of targeted therapeutics (*35*). Recent advancements in the transplantation of porcine kidneys into humans (*36*) have ushered in a new era for organ transplants. Although the clinical success of these groundbreaking xenotransplants has been limited, scientific advancements have moved the field forward. However, substantial challenges remain, particularly in the postoperative stage. Therapeutic drug monitoring and localized delivery are essential to prevent organ rejection and ensure graft survival. The use of mSCRs with mUMRs for targeted drug delivery has clear potential applications in the tailored management of precise posttransplant immunosuppression.

Magnetic actuation is uniquely able to control both tethered and wireless devices across a range of scales. It therefore poses an opportunity for efficient multimodal platforms where the two approaches work together under a shared control (actuation) system to enhance functionality. A magnetic-tip catheter with a rotating magnetic tip has demonstrated its capability of delivering self-expanding stents after clearing intravascular blockages (*37*). Due to the nonmagnetic nature of the stent, a separate procedure is needed to remove or relocate the stent should the situation arise. Catheters with a steerable, separable, and recombinable magnetic tip have shown the navigational benefits of how tethered devices can aid in the advancement of mUMRs (*12*, *38*). These approaches have been primarily demonstrated using either magnetic-tip or nonmagnetic catheters. However, without integrating a fully soft magnetic catheter with navigation-specific parameters, such as tailored magnetic profiles (*16*) or nonuniform magnetic particle distributions (*39*), navigating inside complex anatomical structures remains challenging. Magnetic artificial microtubules, structured microfiber with embedded micromagnets, have demonstrated their ability in guiding swarms of magnetic free-swimming microrobots (*40*). Despite their small size (25 nm), these microcatheters lack the dedicated full shape control offered by mSCRs. Such devices also necessitate the presence of free-swimming microrobots around the microtubules, which can introduce additional risks if these robots, carrying site-specific drugs, travel through nontarget anatomical regions. This poses a potential risk of toxicity along their route to the microtubules. Alternatively, Torlakcik *et al.* (*41*) proposed an mSCR capable of delivering magnetic nanoparticle swarms within the spine. This is done by pumping nanoparticles from a reservoir through the mSCR, demonstrating enhanced precision and reduced delivery time enabled by efficient and targeted delivery (*41*). However, custom continuous magnetization, which is necessary for full shape control and reduced trauma during catheter navigation, as well as the selective retrieval of nonbiocompatible mUMRs, which still make up a majority of presented mUMRs for biomedical applications, remains an open area of research.

**Fig. 1.**
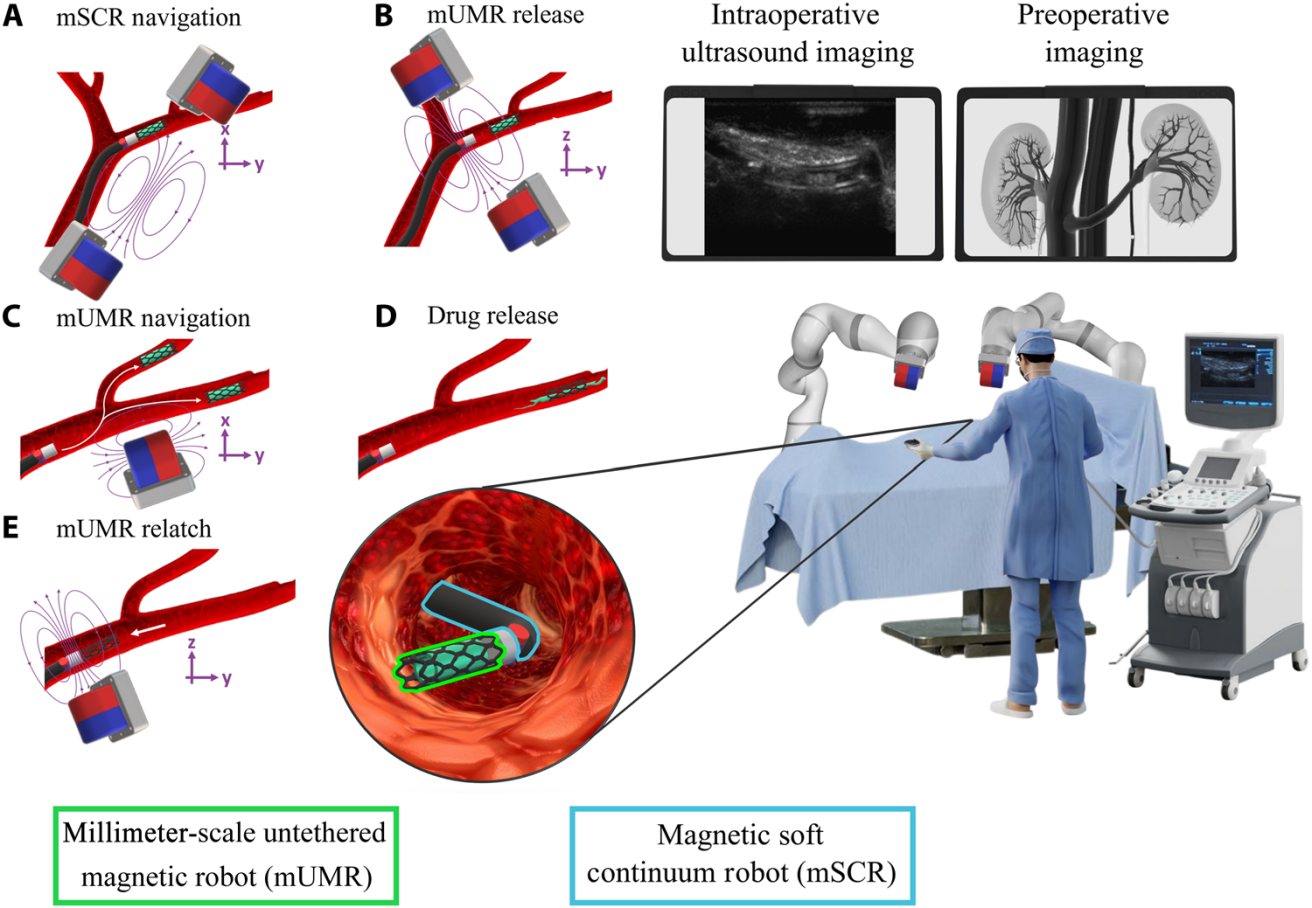
Using mSCRs for the deployment of mUMRs via the magnetic latch. (**A**) Magnetic fields control the mSCR’s shape as it navigates the nonlinear pathways of the kidneys. A drug-doped stent-like mUMR is securely attached to the mSCR using the magnetic latch. (**B**) On the application of the release field, the mUMR is released from the latch. (**C**) The drug-doped mUMR is then independently navigated into the kidney’s narrow vasculature. (**D**) Upon reaching the target location, the mUMR begins site-specific drug delivery. (**E**) After the drug is delivered, the mUMR is reattached to the magnetic latch, and by retracting the mSCR, it is safely removed from the anatomy. The entire procedure is conducted under ultrasound guidance.

To leverage the advantages of mSCRs and mUMRs, we introduce a bespoke, continuously magnetized, magnetic catheter capable of delivering and selectively retrieving millimeter-scale wireless magnetic payloads, as shown in Fig. 1. Although the combination of CRs with untethered micro/nanorobots presents substantial potential, robots of this scale also present complexities that require separate, focused research efforts. Despite other examples of small-scale robots being delivered and retrieved (*12*, *42*), here, we demonstrate a full-length mSCR with a bespoke magnetization delivering and retrieving a drug-doped magnetic payload, i.e., mUMRs, in the ex vivo vasculature scenarios. This is done through the introduction of a magnetic latching mechanism for the delivery and retrieval of mUMRs. This integration of mSCRs and mUMRs improves upon the transportation and delivery of site-specific drugs within the body’s complex, nonlinear pathways. The versatility of our proposed magnetic drug delivery system’s effectiveness is demonstrated using three variations of mUMRs. This includes the stent-like mUMR presented in (*29*), mUMRs of a custom shape made through a spin-coating/laser-cutting process and a dissolvable magnetic drug–based mUMR. Using the dual external permanent magnet (dEPM) presented in (*43*), the efficacy of our proposed system is evaluated through the navigation of an mSCR through a clear silicone phantom, where the mUMRs can then be delivered. The ability to navigate an mSCR to deliver and retrieve mUMRs was demonstrated in a hollow, clear kidney phantom. mUMR navigation under the effect of blood flow was analyzed using a transparent vascular phantom. Given the recent successes in xenotransplantation of porcine kidneys into humans (*36*), we focus our study on the delivery of organ transplantation–related immunosuppressant in the kidney. We demonstrate this by showing the viability of the presented latching mechanism in delivering mUMR in an ex vivo porcine kidney under ultrasound guidance.

## RESULTS

### Magnetic latch design and characterization

**Fig. 2.**
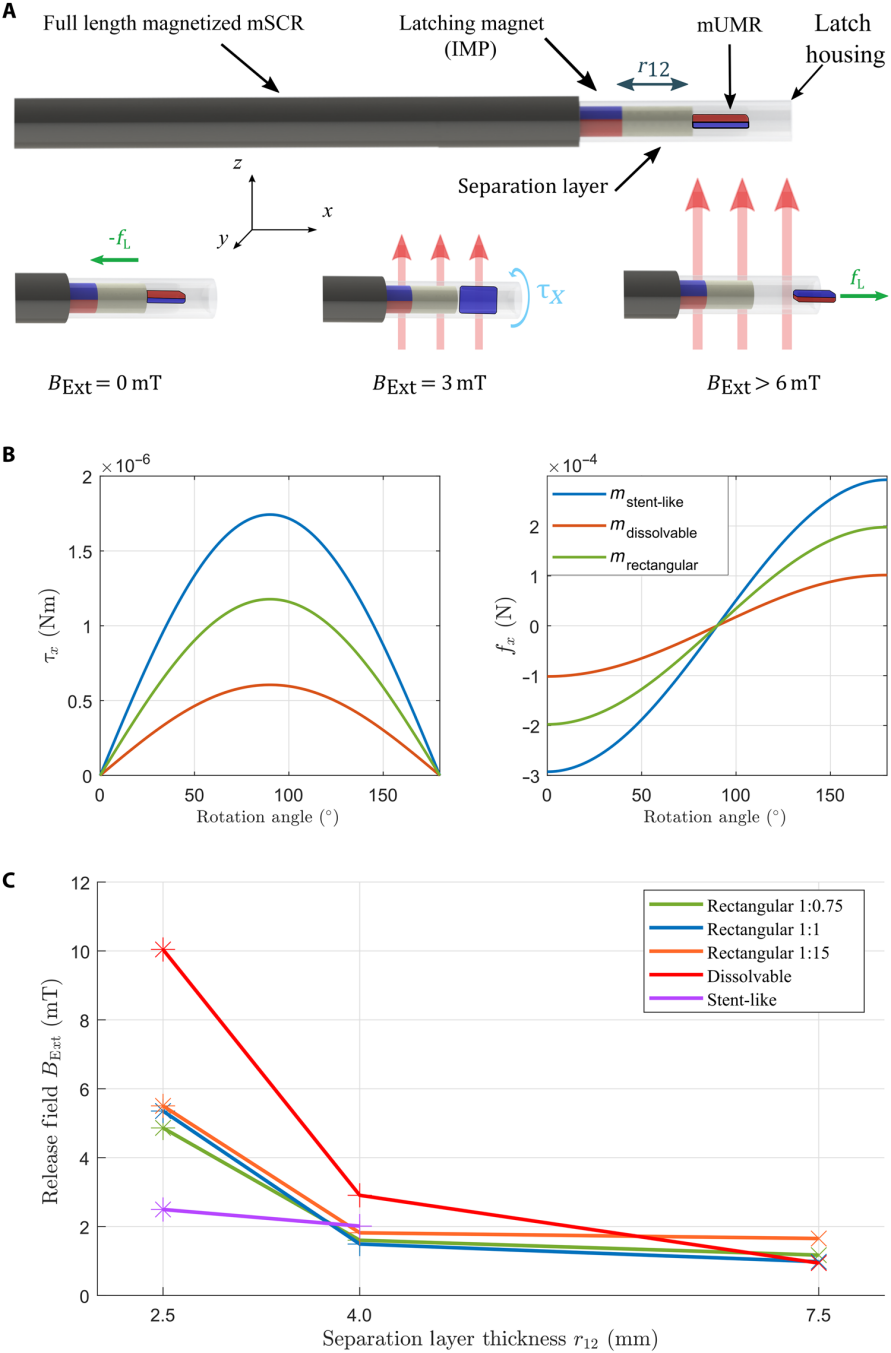
Design and modeling of the magnetic latch. (**A**) Illustration of the magnetic latch attached to the end of an mSCR. The magnetic latch is composed of a latching magnet and a separation layer all confined with a polyurethane tube. Any one of the proposed mUMRs can be inserted within the latch. With no external field $B_{\text{Ext}}$ applied, the mUMR experiences a force, which secures it to the magnetic latch. On the introduction of an external field, the mUMR experiences a torque, and the resulting rotation aligns the poles of the IPM and mUMR causing a force that repels the mUMR out of the latch. (**B**) Calculations expressing how the force and torque experienced by the mUMR changes as it rotates due to the introduction of an external field. Forces and torques are only present along the *x* axis. All other wrench is considered to be negligible. (**C**) The measured external field required to release different mUMRs from the latch with varying separation layer lengths.

The magnetic latch proposed in this article is designed to securely attach and maintain the mUMR throughout the navigation of the mSCR. Once a defined point of deployment is reached, either at the limit of the mSCR’s navigational capability or a desired anatomical region, the mUMR can be magnetically released from the magnetic latch and navigated independently. After the mUMR navigation is complete, it can be magnetically recoupled to the latching mechanism for subsequent retraction and removed from the anatomy via the removal of the mSCR. The magnetic latch consists of a fixed internal permanent magnet (IPM) and a separation layer, all encapsulated within a latch housing polyurethane tube as illustrated in Fig. 2A. When an mUMR is inserted into the latch housing, it experiences a magnetic force attracting it toward the IPM and a magnetic torque rotating it to align its magnetic moment opposite to that of the IPM. In this configuration, the mUMR is securely latched to the mSCR via a force ($-f_{L}$) dependent on the magnetic moments of the IPM and mUMR and their separation distance, defined by the separation layer thickness $r_{12}$.

Releasing the payload from the magnetic latch can be achieved using two methods. First, a magnetic force can be induced on the mUMR that is opposite and greater than $-f_{L}$, separating it from the IPM and expelling it from the latch housing. However, simulation of this scenario (Materials and Methods) demonstrates the requirement of magnetic gradients that exceed the capabilities of currently existing magnetic actuation systems. We therefore consider an alternative unlatching method whereby an external field ($B_{\text{Ext}}$) is applied to rotate the mUMR into the same orientation as the IPM. When the IPM and mUMR have the same orientation, they exert a reciprocal repulsive force expelling the mUMR from the latch housing. The wrench experienced by the mUMR as it is rotated by the external field is illustrated in Fig. 2B. This approach requires a substantially smaller field than using magnetic gradients to separate mUMR from the IPM. This also makes the latch robust to fields that are nonparallel to the magnetization of the IPM, as such fields will not produce the threshold torque required to induce the automatic release of the mUMR from the latch. This de-latching strategy is demonstrated in movie S1.

To explore the relationship between the mUMR, magnetic latch, and the separating layer thickness, the experiment shown in fig. S1A was set up. This consists of magnetic latches with varying separating layer thicknesses placed inside a triaxial Helmholtz coil. The magnetic field along the axis of the IPM (*z* axis) was increased until the mUMR was released from the magnetic latch.

Three styles of mUMRs were chosen due to their variance in applicability. First, the stent-like magnetic soft robot presented in (*29*) was chosen due to its high magnetic moment and capacity to deliver a high concentration of drugs in lumens of varying diameters. The second set of mUMRs consisted of polydimethylsiloxane (PDMS) rectangular elastomer of varying magnetic concentration. These were used to investigate the effect of magnetic moment on the release field. Last, an mUMR composed of gelatin and magnetic particles was used. This was used as a representation of dissolvable drug-doped magnetic materials such as (*44*). This class of mUMRs may be left inside the body to release their payload without the need for retrieval. An overview of the payloads used can be seen in fig. S1C.

The release field required for each mUMR is reported in Fig. 2C and table S1, with the estimated force between the IPM and the different mUMRs detailed in table S2. Each experiment was repeated three times. The magnetic field and gradients were measured using a gradiometer shown in fig. S1B and described in Materials and Methods. The results in Fig. 2C show that, as the separation layer thickness is increased, the field required to release the latch is reduced from 10 to 1 mT for the dissolvable mUMR. This is due to the force between two magnetic objects being inversely proportional to their separation distance to the power of four (*45*). The estimated magnetic torque required to release each mUMR from the magnetic latch is shown in table S3. This was calculated using the approximated magnetic moment and the release field through Eq. 1. Table S3 demonstrates that the torque required to release from the magnetic latch is affected by both the magnetic moment and surface geometries/materials of the mUMRs. Notably, the stent-like mUMR requires less torque for release compared to the dissolvable mUMR and the rectangular mUMR with a 1:1.5 magnetic concentration, despite having a substantially higher magnetic moment. This is attributed to the smoother surface geometry of the stent-like mUMR. In examining rectangular mUMRs with the same geometries but different magnetic concentrations (and therefore, different magnetic moments), we find that a higher magnetic moment requires greater torque for release from the magnetic latch. However, a higher magnetic moment also induces greater opposing torque from the magnetic latch’s IPM. This relationship resulted in a uniform release field for all rectangular mUMR prototypes.

When fields that are not parallel to the IPM are applied, the payload remains securely attached to the latch. The stent-like mUMR was only tested for the first two separation distances. This is because the force between the latch and the stent-like mUMR is insufficient to securely latch at larger separation distances. Consequently, to avoid unintentional release, latching mechanisms with a separation layer thickness of 2.5 mm were used throughout the work presented herein. The effect of having the magnetic latch placed vertically and reattaching the mUMR against gravity was also analyzed and can be seen in movie S1. This illustrates how the magnetic latch successfully retrieves mUMRs even when gravity is a factor. Therefore, influence of gravity is negligible in the effectiveness of the magnetic latch’s retrieval capability for the chosen mUMRs.

### mSCR design for renal navigation

**Fig. 3.**
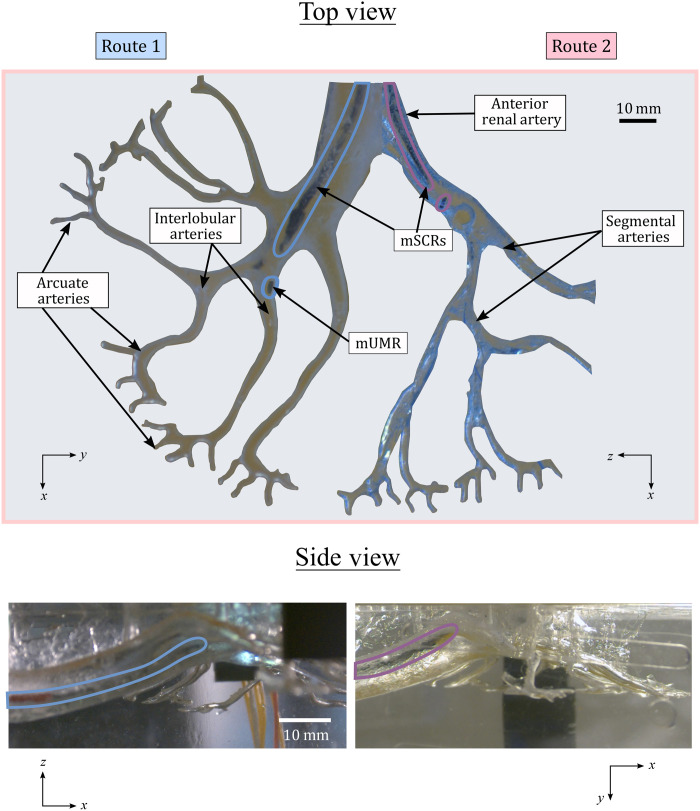
Navigation of an mSCR in a soft kidney phantom. Two route-specific mSCRs were navigated within a soft kidney phantom. They were introduced through the anterior renal artery, and 3D navigation was performed until the lumen diameter narrowed to match the mSCRs. At this point, a release field was applied to detach the mUMR, allowing for independent, untethered navigation from the interlobular arteries into the arcuate arteries.

Despite the diversity of potential applications of the presented work, we focus on the robotic delivery and subsequent navigation of mUMRs for targeted drug release in the kidney. The procedural approach focused on arterial navigation, enabling direct drug absorption to enhance renal function and reduce inflammation and oxidative stress. We designed two route-specific mSCRs, each with a diameter of 1.5 mm and a length of 40 mm. These mSCRs were intended to navigate through the two segmental arteries highlighted in Fig. 3. This design allows for the delivery of mUMRs to the interlobar arteries of the kidney, where their untethered navigation begins. After segmenting the volumetric data to extract the two paths of interest, we used the established pseudorigid-link elastomechanical optimization detailed in (*46*) to obtain path-specific magnetization patterns and associated actuating magnetic fields for the two mSCRs.

Consistent with (*17*) and (*16*), we assume planar deformation, meaning that any curvilinear path exhibits a principal plane that minimizes out-of-plane deformation. Therefore, we only impart magnetization in this same plane. For the navigations selected in this study, using spatial data extracted from the anatomical computed tomography (CT) scan, rotating our catheter into this plane results in maxima of 2.3 and 4.3% (as a percentage of path length) out-of-plane deformation for navigations 1 and 2, respectively. Furthermore, the tip magnet, which actuates the latching system, lies perpetually orthogonal to the plane of deformation such that it does not affect optimization assumptions but would, in free space, generate some out-of-plane deformation. For the two paths of interest, the maximum out-of-plane deformation was measured at 4.85 mm, with an average of 3.00 mm across all planned paths. The out-of-plane deformation for each section of the planned path is detailed in table S4, and additional information on how this deformation was characterized is provided in Materials and Methods. The planar assumption, the tip-mounted latch magnet, and further standard modeling assumptions (e.g., linear elasticity, zero demagnetization) result in some degree of simulation error, which was counteracted via the experimental tuning of the required actuating field.

For the navigation of route 1, the planar plane of navigation is the *x*-*y* plane of the global reference frame shown in fig. S2. To navigate route 2, the phantom was rotated by 90° about the *x* axis. Repositioning of the patient is commonly practiced as it can facilitate access to anatomical locations (*47*). Using these orientations, the planar magnetization optimization assumptions stated above are satisfied for mSCR navigation through both pathways. The magnetization profiles for each segmented pathway can be seen in fig. S3 while the magnetic fields required for each stage of the navigation procedure can be found in table S5.

### Magnetic path planning and mSCR navigation

Actuation of the mSCRs and deployment and navigation of the mUMRs were performed using the dEPM platform. The dEPM platform has demonstrated the capability to independently actuate up to eight magnetic DoFs at a medically relevant scale (*43*). Its large workspace and multi-DoF capabilities are essential for controlling the shape of the mSCR and for releasing and navigating the mUMR. However, during transitions between EPM poses, the dEPM platform can inadvertently produce unwanted magnetic fields or gradients. Such unplanned EPM transitions may result in EPM-to-EPM collisions and unintended actuation of the mSCR. To prevent unintended actuation of the mSCR and accidental activation of the release field during mSCR navigation, we use the hybrid trajectory planner proposed in (*48*). By leveraging the previously discussed mSCR magnetization optimization, we ensure that the magnetic latch release field is not part of the mSCR navigation, allowing the hybrid trajectory planner to effectively prevent unintentional mUMR release. This planner was used to determine the EPM paths necessary for navigation within the kidney phantom, preventing unintentional release of the mUMR. Navigation of the mSCRs within the kidney phantom was repeated three times for each path and is shown in Fig. 3.

As shown previously in Fig. 2, a field below −5.5 mT is enough to actuate the magnetic latch. The polarity of this threshold value is relative to the direction of magnetization of the IPM. When not implementing the hybrid trajectory planner, a field of −20.5 mT along the *z* axis was inadvertently actuated (373% of the required actuation field). This led to the unintentional release of the mUMR and is highlighted in fig. S4 and movie S2. With the use of the hybrid trajectory planner, the actuation of the release field was kept to −0.83 mT (15% of the required actuation field) during the navigation process. This ensured that navigation of the mSCR was performed with the mUMR securely attached within the magnetic latch. Once the mSCR reached the end of its navigation path, the mUMR was deployed by actuation of the release field and navigated independently.

### Integrated mSCR navigation with mUMR deployment and retrieval

Using the same segmentation data as the soft kidney phantom depicted in Fig. 3, we three-dimensionally (3D) printed a hollow kidney phantom using Clear Resin v5 (Form 4, Formlabs, USA). The phantom was filled with water mixed with red dye to enhance visibility. Using the hybrid trajectory planner and magnetic fields generated through the aforementioned optimization process, an mSCR with a 1:15 rectangular mUMR, attached via a magnetic latch, was navigated through route 1 of the phantom. Upon completing the mSCR navigation, the surgeon took control of the dEPM platform using a game controller (Logitech wireless gamepad F710, Logitech, Switzerland). At the end of route 1, two accessible interlobular arteries were identified, providing access to three separate arcuate arteries, as indicated in Fig. 3 and fig. S3.

The surgeon guided the mSCR into each interlobular artery in separate experimental repetitions. As the diameters of the interlobular arteries matched the mSCR’s diameter, the surgeon released the mUMR, which was independently navigated to the distal ends of the phantom. Subsequently, the mUMR was maneuvered back to the mSCR and securely reattached via the magnetic latch, allowing for their joint removal from the anatomical model. This procedure was repeated for the two interlobular arteries accessible through route 1 to access all three arcuate arteries. This process is illustrated in movie S3.

### Influence of blood flow on mUMR navigation and retrieval

**Fig. 4.**
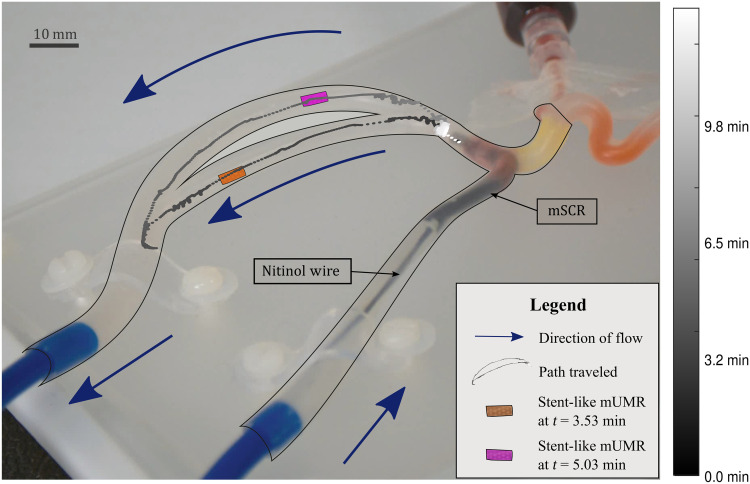
Deployment, navigation, and retrieval of mUMRs against flow. The mSCR was navigated to a shunt within the vascular phantom. To simulate the effect of blood flow, water was pumped through the phantom in the directions indicated by the blue arrows. The mUMR was magnetically released from the latch and navigated around the phantom. This process was repeated three times in a clockwise direction, as shown, and another three times in the opposite direction. Subsequently, the mUMR was reattached to the latch while opposing the flow. The colormap illustrates the average position of the stent-like robot during clockwise navigations.

Once the mSCRs have been successfully navigated, the mUMRs can be deployed and independently maneuvered. To evaluate the effect of blood flow on the deployment, navigation, and retrieval of the mUMR, the vascular setup shown in Fig. 4 was used. This setup comprises a vascular phantom (Trandomed 3D, Ningbo, China) arranged in a circular loop with an internal diameter of 1.5 mm and a circulating flow (Flowbox One pulsatile flow machine, HumanX Medical Simulators, Florida, USA). Water was pumped through the phantom at a rate of 1.3 liters/min to mimic the blood flow in a human kidney (*49*). This experiment tests the hypothesis that the mUMRs can be unlatched against the flow and navigated both with and against it. Because the goal of the dissolvable mUMR is to replicate the behavior of biocompatible mUMRs, which do not require retrieval, in this experiment, we used the stent-like mUMR and the rectangular mUMR, both requiring removal from the anatomy after navigation.

Both classes of mUMRs were released and navigated three times in a clockwise direction and three times in a counterclockwise direction; being relatched each time, as shown in movie S4. Figure 4 shows the mean path taken for the stent-like mUMR navigated in the clockwise direction. The navigational process was divided into three stages: release from the latch, navigation around the phantom, and reattachment to the magnetic latch. The mean time taken for each section is listed in table S6, while the average norm of the magnetic gradient used for each stage is shown in fig. S5. Notably, the time taken for reattachment had the most variability between experiments (±10.07 min for the rectangular mUMR in the clockwise direction and ±9.30 min for the stent-like mUMR in the anticlockwise). This is due to the alignment challenges posed by relying on a single camera view positioned over the phantom. Despite having comparable navigation times in each phase of the navigation, the rectangular mUMR was slightly quicker. This may be related to the stent-like mUMR’s larger surface potentially leading to a higher shape-induced flow resistance. When considering the magnetic gradients applied, the stent-like robot generally required a lower magnetic gradient during navigation. In the clockwise navigation scenario, 32% of the magnetic gradients used exceeded 1500 mT/m for the stent-like mUMR, compared to 38% for the rectangular mUMR. In a similar manner, for the anticlockwise navigation, gradients ranging from 500 to 1500 mT/m constituted 32 and 48% of the total gradients for the stent-like and rectangular mUMRs, respectively. This indicates that higher overall magnetic gradients were required to generate the necessary force for navigating the rectangular mUMR compared to the stent-like mUMR. This stems from the stent-like mUMR’s higher magnetic moment and circular shape, enhancing its navigational capabilities. In addition, the effect of the simulated blood flow overcoming the magnetic coupling between the mUMR and the external magnetic field can be seen in movie S4.

### Drug delivery using mUMRs

To evaluate the potential for drug delivery using the presented mUMRs, each mUMR design was coated with a payload replicating a mock drug composed of red food dye and gelatin. The coated mUMRs were placed in the phantom depicted in fig. S6A, which contained water at 18°C. All the mUMRs were coated with 1 mg of the mock drug. The volume of the drug released was quantified by measuring the red pixel intensity over 30 min, as shown in fig. S6B. Analysis of these results indicates that the stent-like mUMR released the red dye most rapidly, with approximately double the amount of dye released after 30 min. This is due to the high surface area of the stent-like mUMR, which facilitates more rapid drug release. To demonstrate how this response can be adjusted for desired drug-release profiles, one of the mock drug-coated stent-like mUMRs was further coated in gelatin, which substantially altered the release rate of the red dye. Figure S6B shows how the release of the red dye experiences a logarithmic growth. This further emphasizes the need for delivery of site-specific drugs directly at their intended location.

### Ultrasound-guided mUMR delivery and retrieval in ex vivo porcine kidney

**Fig. 5.**
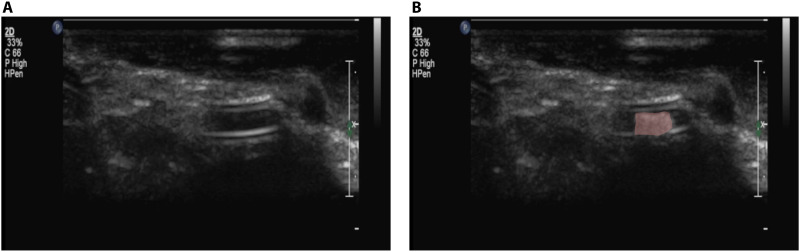
Navigation of mUMR in a porcine model under ultrasound guidance. (**A**) A stent-like mUMR securely attached to the mSCR using the magnetic latch and viewed under ultrasound imaging. (**B**) The stent-like mUMR (highlighted in red) is then independently navigated into the kidney’s narrow vasculature by the surgeon. After the mUMR has released its payload, it is reattached to the magnetic latch, and by retracting the mSCR removed from the anatomy.

To assess the clinical efficacy of the proposed latch mechanism, we demonstrated the release and reattachment of an mUMR against blood flow within an ex vivo porcine kidney under ultrasound guidance. Ultrasound was selected for its noninvasive and nonionizing properties, allowing for real-time tracking throughout the procedure. The experimental setup, shown in fig. S7A, used a Philips L9-3 probe connected to an iU22 ultrasound machine (Philips, Amsterdam, Netherlands) to scan the porcine kidney and provide a live feed to the surgeon. The surgeon controlled the position of an EPM and navigated the stent-like mUMR using a Logitech wireless gamepad F710 (Logitech, Switzerland) as shown in Fig. 5. This mUMR was chosen for its superior visibility under ultrasound, with comparisons to other mUMRs available in fig. S7B.

The stent-like mUMR was successfully released and reattached to the latch three times, with an average process time of 20.42 ± 17.34 min. This process time includes the duration for the stent-like mUMR to be released from the magnetic latch, navigated through the ex vivo porcine kidney model, reattached to the magnetic latch, and subsequently removed from the anatomical model. This procedure was repeated three times using the same stent-like mUMR in the same ex vivo kidney model. This experiment effectively demonstrates the procedure of navigating an mSCR into anatomical structures, releasing an mUMR to independently deliver its payload, and can be seen in movie S5. The effects of not having sufficient coupling between the stent-like mUMR and the EPM are also demonstrated in this video.

## DISCUSSION

In the presented article, we discuss targeted drug delivery under magnetic guidance in the context of organ transplantation. Immunosuppressants like cyclosporine (CsA) prevent allograft rejection in organ transplantation. However, CsA has a narrow therapeutic window and presents nephrotoxicity as a substantial side effect (*50*). Sitagliptin and hesperidin have demonstrated potential in mitigating CsA-induced nephrotoxicity but require precise delivery methods.

We present a full shape forming mSCR with an integrated magnetic latch for mUMR transportation and delivery. Using a magnetization profile along the whole length of the mSCR allows for navigation through complex anatomy, while the magnetic latch allows successful on-demand deployment of payloads in the vicinity to their intended site. The magnetic nature of the latch, along with a tunable separation distance, makes it universally applicable to mUMRs with a permanent magnetization, a common approach for microrobot design (*51*, *52*), and is therefore agnostic from the mUMR actuation strategy. In addition, the incorporation of an IPM within the latch allows for the selective retrieval and removal of the mUMR. This contrasts with previous techniques, which used a channel running the length of the mSCR to retrieve swarms of mUMRs via suction (*41*), and therefore, do not offer the same level of selectivity as the proposed system.

The universal applicability of the presented latching mechanism is further supported by the variety of mUMRs used. The stent-like mUMR has already demonstrated its medical potentials in procedures such as removing blood clots and diverting the blood flow for brain aneurysms (*29*). This payload was chosen to represent similar mUMRs with high magnetic moments and substantial drug-carrying capacity. This, in combination with the emergence of flexible, digitally controlled deposition technologies (*53*) and programmable spatial magnetization methods (*54*) could enable a new class of patient-specific drug delivery methods. The rectangular mUMRs used in this article were selected to represent emerging mUMRs that can be easily infused with medications. However, this class of mUMR experiences low visibility under ultrasound. The incorporation of microbubbles within these mUMRs has been shown to enhance their contrast during ultrasound imaging (*55*, *56*). In addition, we highlight the magnetic latch’s capability in deploying drug-infused dissolvable mUMRs. The development of biocompatible magnetic materials, such as samarium-cobalt (SmCo_5_) magnets, has facilitated the creation of this new class of mUMR (*57*). These payloads enable high-concentration drug delivery. Once released from the latching mechanism and navigated to the target location, the drug-doped mixture dissolves, releasing its payload into the surrounding tissues. However, this class of mUMRs typically exhibits a low magnetic moment.

In an integrated experiment, we demonstrated the magnetic latch’s potential for delivering and retrieving mUMRs within realistic anatomical models. An mSCR with customized magnetization was successfully navigated through a 3D-printed kidney phantom. Upon reaching the predetermined interlobular artery, where the artery diameter matched that of the mSCR, the magnetic latch was activated to release the mUMR. The mUMR was then independently navigated to the arcuate arteries before being directed back to the magnetic latch for retrieval and subsequent removal from the kidney phantom. As shown in movie S3, some unintended movement of the mSCR tip, containing the magnetic latch, was observed during mUMR control and navigation. This undesired motion could be mainly attributed to the fact that release and independent control of the mUMR was performed through manual control of the EPMs’ position using a game controller. Future work will therefore focus on developing algorithms for automated release and navigation of the mUMR. These will help to reduce the tortuous movement of the mSCR and shorten the time required for mUMR navigation and reattachment. These algorithms along with the integration of soft anchoring mechanisms, such as those proposed in (*58*, *59*), will help stabilize the mSCR during mUMR navigation.

As highlighted when navigating mUMRs against blood flow, re-attachment of the mUMR required a varying amount of time (table S6). This is due to the precise 3D alignment required between the mUMR and the latch along with the limited views presented to the surgeon. We acknowledge that these constraints limit practical applicability at this stage; however, future work aims to address this challenge through the integration of shape-sensing technologies in the mSCR, such as Fiber Bragg grating, and using magnetic localization techniques to accurately track the position of the mUMRs. In addition to reducing relatching time, these improvements would facilitate the development of closed-loop control systems, which do not require optical feedback. Such systems could minimize the risk of the mUMR losing magnetic coupling with the EPM and being inadvertently carried away by blood flow as seen in movie S5. To further reduce the variability in latching times, a potential future enhancement could involve incorporating passive alignment and guiding structures within the magnetic latch. These features could assist in guiding the mUMR back into the magnetic latch for reattachment, thereby enhancing the system’s robustness. Due to the fundamental relationship between the orientation of the IPM and the polarity of the release field applied, the orientation of the IPM was verified before insertion into the relevant phantoms or ex vivo models. Future work may involve the incorporation of ultrasound contrast media, such as microbubbles, to help identify the IPM’s orientation when this cannot be done visually.

Another area for potential future work involves incorporating planning algorithms that account for the body’s dynamic nature, such as breathing (*60*) and cardiac pulsation (*61*). These directions are essential to bridge the gap between simplified phantom studies and clinically relevant scenarios. Using these algorithms could ensure that undesired forces from the anatomy acting on the magnetic latch are accounted for. A further improvement involves modifying the magnetic latch to control multiple mUMRs, using a similar control methodology to that presented in (*30*).

In contrast to other mUMR deployment technologies, such as hydraulic release (*41*), which provides an elegant solution for single-use delivery, the magnetic latch enables selective retrieval and repositioning, addressing the need to safely remove mUMRs that often contain nonbiocompatible materials. This functionality makes the proposed system particularly advantageous for procedures where retrieval is required.

## MATERIALS AND METHODS

### Magnetic latch design and modelling

When designing the magnetic latch, we must consider the effects of magnetic materials within a background field. An object with magnetic moment $m\in {\mathbb{R}}^{3}$ inside an applied field $B\in {\mathbb{R}}^{3}$ experiences a magnetic torque $\tau \in {\mathbb{R}}^{3}$, which can be defined as

τ=m×B (1)

Using the magnetic dipole model and neglecting higher-order terms [an accurate assumption as when the separation distance is greater than the magnet’s diameter, the error is just below 5% (*62*)], the torque between two magnetic objects can be expanded as (*45*)

$$\tau =S\left\{m_{2}\right\}\left[\frac{\mu_{0}}{4\pi {|r_{12}|}^{3}}\left(3{\hat{r}}_{12}{\hat{r}}_{12}^{T}-{𝕀}_{3}\right)\right]m_{1}$$ (2)

where $\mu_{0}$ is the permeability of free space, $r_{12}\in {\mathbb{R}}^{3}$ is the distance between the two magnetic objects, $I\in {\mathbb{R}}^{3\times 3}$ is the identity matrix, S{·} is the skew-symmetric matrix, ∣·∣ is the Euclidean norm, and $\hat{\cdot }=\frac{\cdot }{|\cdot |}$ is a unit vector. In the presence of a magnetic gradient, a magnetic object experiences a force $f\in {\mathbb{R}}^{3}$, such that

f=(m.∇)B (3)

where ∇ is the gradient operator. Similarly, Abbott *et al.* (*45*) show that the induced magnetic force between two objects can be expanded using the dipole model as

$$\begin{array}{cc} f & =\frac{3\mu_{0}}{4\pi {|r_{12}|}^{4}}\left\{\left({\hat{r}}_{12}^{T}m_{2}\right)m_{1}+\left({\hat{r}}_{12}^{T}m_{1}\right)m_{2}\right.\\ & +\left.\left[m_{1}^{T}m_{2}-5\left({\hat{r}}_{12}^{T}m_{1}\right)\left({\hat{r}}_{12}^{T}m_{2}\right)\right]{\hat{r}}_{12}\right\} \end{array}$$ (4)

When an mUMR is inserted into the latch, their interactive force is governed by Eq. 4, with $m_{1}$ being the magnetic moment of the latching magnet, $m_{2}$ the magnetic moment of the mUMR, and $r_{12}$ the thickness of the separation layer. To release the mUMR from the latch, an external magnetic gradient $G\in {\mathbb{R}}^{6}$ could theoretically be applied. This gradient exerts a force on the mUMR that exceeds the force holding the mUMR to the latch ($f_{\text{latch}}$). The required gradient can be calculated as

$$G={m_{2}}^{†}.f_{\text{latch}}$$ (5)

where ·^†^ is the Moore-Penrose inverse. This would require unfeasibly high gradients to separate the mUMR from the latch. For the stent-like mUMR at a separation distance of 4 mm, a gradient of 5.625 T/m would be needed, which is higher than that which most magnetic actuation systems can produce at a medically relevant scale (*63*). Therefore, we instead use a torque-based approach to release the mUMR from the latch. In its latched state, $m_{1}$ and $m_{2}$ are antiparallel such that opposing poles are neighboring. Therefore

$$\hat{m_{1}}=-\hat{m_{2}}$$ (6)

By applying a torque, the orientation of the mUMR can be flipped such that $\hat{m_{1}}=\hat{m_{2}}$. This will repel the mUMR out of the latch with a force dictated by Eq. 4. Using this approach, the torque required is dependent on the magnetic latching force and the friction between the mUMR and the latch housing. For the mUMRs presented here, the magnetic field required to generate the required torque ranges from 2 to 10 mT, as derived experimentally in Fig. 2, which is easily achievable by most commercial magnetic actuation systems.

### mUMR magnetic moment estimation

The magnetic moment of the mUMRs determines the force and torque between latch and mUMR according to Eq. 4. While the magnetic moment of the stent-like mUMR is known (*29*), the magnetic moment of the rectangular and dissolvable mUMRs was estimated as follows. Given known mass (±0.1 mg; Sartorius Analytical Balance, Sartorius AG, Germany), the mUMRs were placed on the characterization setup shown in fig. S8. A motorized linear stage (NRT150/M, Thorlabs, Inc., USA) vertically lowered an axially magnetized cube magnet (length, diameter, and height of 25.4 mm; N42, First4Magnets, UK) until the gravitational force of the mUMR was overcome by the attractive force of the EPM. At this distance, between the EPM and the bottom of the stage ($r_{\text{char}}$), the force between the EPM and the mUMR is equal to the weight of the mUMR multiplied by gravity. Therefore, Eq. 4 can be solved to find the magnetic moment of the mUMR. This procedure was repeated five times per mUMR. The resulting magnetic moments appear in table S1.

### mSCR fabrication

To fabricate the mSCRs, a magnetic slurry was made by mixing NdFeB microparticles (5-μm diameter; MQFP-B+, Magnequench GmbH, Germany) with a silicone-based elastomer (Dragon Skin 30, Smooth-On, Inc., USA) in a 1.5:1 mass ratio. The magnetic slurry was degassed and mixed in a high vacuum mixer (ARV-310, THINKYMIXER, Japan) at 1400 rpm, 20.0 kPa for 90 s. The degassed magnetic material was injected into a 3D-printed (Tough PLA, Ultimaker S5, USA) 50-mm long, 1.5-mm diameter cylindrical mold. This was cured in an ultraviolet (UV) oven for 30 min at 40°C (Form Cure, Formlabs, USA). The unmagnetized mSCRs were then secured into 3D-printed (Tough PLA, Ultimaker S5, USA) magnetization trays [as in (*64*)], containing the needed magnetic profiles. Each mSCR was then magnetized by subjecting it to a uniform saturating magnetic field of 4.644 T (ASCIM-10-30, ASC Scientific, USA). To enable insertion of the mSCR into the soft kidney and vascular phantom, a 40-mm long, 0.75-mm diameter Nitinol wire was attached to the mSCR. The base of the Nitinol wire was attached to a Bowden cable passing through a mechanical introducer (Hybrid Stepper Motor MT-1703HSM168RE, MOTECH MOTOR Co. LTD, China). This was used to introduce the mSCR into the kidney phantom at a speed of 1 mm/s.

The magnetic profiles of the mSCRs were calculated using the technique described in (*46*). The wrench on any pseudolink is the sum of all distal wrenches, themselves a function of deformed magnetization and applied actuating field. This aggregate wrench is balanced against the elasticity of the material in the form of a 3D pseudojoint. Using this simulation, we optimize via gradient descent to determine magnetization profiles and applied fields that will create deformations in agreement with the predefined anatomical pathways (in the form of joint angles) at sequential time-steps to achieve follow-the-leader navigation. The mSCRs were accordingly magnetized by subjecting them to a uniform saturating magnetic field of 4.644 T (ASCIM-10-30, ASC Scientific, USA).

### Magnetic latch fabrication

The magnetic latch consists of a latching magnet and separation layer all contained with a latch housing. A 1.5-mm diameter polyurethane tube (Nordson Medical, USA) was chosen for the latch housing due to its low friction coefficient, allowing for smooth movement of the mUMR. Two NdFeB N45 magnets (Supermagnete, Germany) with a height of 0.5 mm and diameter of 1.5 mm were axially stacked to formulate the IPM and inserted into the latch housing. A separation layer was subsequently created by inserting a 3D-printed (Rigid 4000 V1, Formlabs III, USA) 1-mm diameter cylinder of desired thickness into the latch housing on top of the IPM. With the latch housing placed in a vertical position with the IPM at the bottom, a fast cure flexible epoxy adhesive (IRS 2126, Intertronics, UK) was then injected from the bottom to coat the latching magnet and the separation tube. Before the flexible epoxy was given time to cure, the mSCR was inserted into the polyurethane tube to securely attach the latching mechanism to the mSCR. Mechanical analysis of the bonding agent used can be found in note S1. After allowing the epoxy to cure for 2 hours as per the manufacture’s guidelines, the polyurethane tube was cut to the desired length (tunable parameter, which is mUMR specific). This length ensures that upon release from the magnetic latch, the mUMR travels linearly outward and does not rotate to reattach itself to the latch IPM or mSCR. The combination of this tube length along with the stronger magnetic field generated by the EPM, compared to that of the IPM, ensures that the mUMR is guided smoothly out of the magnetic latch and follows the path dictated by the EPM’s position.

### Out-of-plane deformation

To characterize the out-of-plane deformation caused by the latch IPM, which is orthogonal to the plane of magnetization of the mSCRs, the mSCRs were set up in a free-space environment, as shown in fig. S9. The triaxial Helmholtz coil depicted in fig. S9A was used to generate the magnetic fields necessary for navigating each mSCR through its designated path (table S5). A camera positioned below the mSCR measured any out-of-plane deformation, with results detailed in table S3. Although mSCRs are rarely used in free-space environments and are generally constrained by the surrounding anatomy, long term, we still aim to reduce this error by integrating a closed-loop control system.

### Fabrication of rectangular mUMRs

First, PDMS (Sylgard 184, Dow Chemicals, USA) was prepared by mixing the crosslinking agent with the base in 1:10 ratio as per supplier guidance. NdFeB powder (5-μm diameter; MQFP-B+, Magnequench GmbH, Germany) was then added to give the desired ratio. Here, three ratios of elastomer to NdFeB were prepared, 1:0.75, 1:1, and 1:1.5. The composite was then mixed with a planetary mixer-degasser (Thinky ARE-250, Intertronics, UK), mixing, and defoaming for 2 min at 1500 rpm each. While degassing, a 150-mm silicon wafer was coated with a release agent (Easy Release 200, Bentley Advanced Materials, UK). Once prepared, the NdFeB elastomer composite was spin-coated onto the silicon wafer at 200 rpm for 30 s. Subsequently, the layer was cured at 100°C for 1 hour, as per supplier guidance. Once cured, the mUMRs were laser cut using a high-resolution UV laser system (Meta-C UV 3 W, Lotus Laser Systems, UK), and could be removed from the silicon wafer with tweezers.

### Stent-like soft magnetic robot fabrication

A positive model of the stent-like mUMR was 3D printed (IPQ, Nanoscribe GmbH, Germany). This was used to create a negative mold of the robot using PDMS (Sylgard 184, Dow Chemicals, USA). A magnetic slurry composed of PDMS and NdFeB powder (5-μm diameter; MQFP-B+, Magnequench GmbH, Germany) mixed in a 1:4 mass ratio was made. The mixed polymer was cast into the negative PDMS mold and cured at 85°C for 7 hours. The sample was then magnetized using a vibrating sample magnetometer (VSM, EZ7, Microsense, USA) with a 1.8-T uniform magnetic field. The stent-like mUMR was demolded to produce the final specimen. The mUMRs where magnetized along their *z* axis by applying a 2.7-T uniform field (DXMM-20C20 Magnetizer, Dexing Magnet Tech. Co, Limited, China).

### Gelatin dissolvable mUMR fabrication

A mixture of gelatin, NdFeB, pigment, and water with a mass ratio of 1:1:0.5:5 was prepared. The gelatin (150 Bloom, Scientific Laboratory Supplies, UK) was combined with NdFeB (5-μm diameter, MQFP-B+, Magnequench GmbH, Germany) microparticles and red pigment (Wurth Essenzenfabrik GmbH, Austria) and mixed in a beaker with deionized cold water. The mixture was allowed to bloom for 30 s before further processing. Dispersion homogeneity was ensured using a hot plate magnetic stirrer (Heidolph MR-Hei Standard, Germany), set to a plate temperature of 60°C and a speed of 800 rpm. The resulting mixture was then poured into a glass petri dish and placed in cold water in a refrigerator. This quenching process froze the mixture and prevented sedimentation during cooling. Once solidified, the mUMRs were cut, transferred to a 24-well plate, and stored in a refrigerator at 4°C until experimentation. Before conducting any related experiments, these mUMRs where magnetized along their *z* axis by applying a 2.7-T uniform field (DXMM-20C20 Magnetizer, Dexing Magnet Tech. Co, Limited, China).

### Mock drug deposition on mUMRs

Using the same procedure as when making the gelatin-based mUMRs, a gelatin aqueous solution with red pigment (Wurth Essenzenfabrik GmbH, Austria) was prepared and dispensed using a micropipette to achieve uniform microdroplets. A total of 1 mg of colored microdroplets was added to the stent-like and rectangular mUMRs. The gelatin coated mUMRs were subsequently stored in a refrigerator at 4°C. Once the gelatin-color mixture has cured, a layer of color-free gelatin aqueous solution could be applied to the mUMRs to alter the rate of red dye released over time.

Following the drug release demonstration shown in fig. S6, the recorded videos were processed using MATLAB (R2023a) to analyze and compare the efficiency of drug release in relation to the mUMR over time. A custom script was developed to calculate the ratio of red intensity by measuring the red channel values relative to the number of red pixels in each video frame. To facilitate comparison across all videos, the highest recorded value was identified and stored as the global maximum for normalization purposes. This approach enables a clear and consistent representation of the drug release kinetics in an aqueous medium.

### Soft kidney phantom fabrication and setup

To demonstrate the navigational capabilities of the mSCRs, a soft kidney phantom was made. A 3D representation of the renal artery was obtained by segmenting CT angiography data (https://cults3d.com/en/3d-model/various/kidney) for a healthy human kidney. The volumetric data of the renal arteries were 3D printed (Rigid 4000 V1, Formlabs III, USA). The 3D-printed part was suspended in a 120 mm–by–124 mm acrylic box. Silicone (Ecoflex Gel, Smooth-On, Inc., USA) mixed with cure retarder (SLO-JO, Smooth-On, Inc., USA) with a ratio of 5% by weight was poured into the acrylic box. The now submerged 3D-printed part was placed within a vacuum chamber (Renishaw Vario 5/01 vacuum casting machine, Renishaw, UK) to remove any air bubbles before curing the silicone mixture at 40°C for 40 min (Genlab Prime, Genlab, UK). Once the silicone had cured, the 3D-printed part was removed from the acrylic box, leaving behind a clear silicone phantom of the renal arteries.

### The dEPM platform

The dEPM platform is made up of two axially magnetized, cylindrical N52 EPMs (101.6 mm diameter and length), each mounted on a KUKA LBR iiwa14 (KUKA, Germany) serial robotic manipulator. This platform, initially presented in (*43*), is capable of safely generating fields of up to 200 mT and magnetic gradients of up to 500 mT/m. The dEPM platform is able to produce different combinations of magnetic fields and gradients, with 81.1% of random field and gradient combinations produced with less than 1% error. The dEPM system is also equipped with a four-camera optical tracking system (OptiTrack, NaturalPoint, Inc., USA). The optical tracking system was used for a calibration procedure. Here, optical tracker markers are used to relay the position of an object of interest (for example, the kidney phantom) to the dEPM platform as seen in fig. S2. A further explanation of the calibration procedure can be found in (*43*).

### Preparation of porcine kidney for ex vivo experiments

Porcine retroperitoneal organs, including the kidneys, pancreas, renal arteries, and ureter, were procured from a slaughterhouse in accordance with the UK Department of Health and Social Care guidelines for the handling and transport of tissue. Fresh organs were collected and immediately placed in a plastic bag on ice for transport in a dedicated medical sample carrier (UN 3373, MedDXTAINER, UK). The carrier was sealed until delivery to our mock operating theater.

In the operating theater, the renal artery and kidneys were isolated. All vessel branches and the posterior division of the renal artery were ligated to minimize saline solution loss. The kidney was then positioned on a plastic medical tray and connected to the pump perfusion system by cannulating the proximal end of the renal artery and the distal ends of the ureter. Cannulation was achieved by suturing a synthetic “Y” graft to the anterior division of the renal artery, using a technique similar to that used in coronary artery bypass grafting. This ensured a secure connection to the perfusion system and enabled the insertion of the mSCR.

Between experiments, tissue storage was maintained in a dedicated refrigerator at 4°C, avoiding freezing or any alteration of specimen properties. During the surgical procedures, the most common perfusion solution is 0.85% NaCl saline. However, as recommended by the World Health Organization (*65*), it is standard laboratory practice to use a buffered saline solution to maintain a pH closer to physiological levels. The solution was prepared by dissolving buffered saline tablets in distilled water and added to the reservoir.

### Magnetic field and gradient measurement

Magnetic fields and gradients were measured using a custom-made magnetic gradiometer. The gradiometer is made out of three three-axis magnetic field sensors (AK09973D, Asahi-Kasei, Japan) laid out as shown in fig. S11. The position of sensor U2 is considered to be the center of the magnetic gradiometer; therefore, the magnetic field is simply the fields recorded by this sensor. Through knowing the distance between the three sensors (∆x and ∆y), the independent components of the magnetic gradient (G) could be estimated as follows

$$G=\left(\frac{B_{x1}-B_{x2}}{\Delta x},\frac{B_{y1}-B_{y2}}{\Delta x},\frac{B_{z1}-B_{z2}}{\Delta x},\frac{B_{y3}-B_{y2}}{\Delta y},\frac{B_{z3}-B_{z2}}{\Delta y}\right)$$ (7)

where the subscript $\cdot_{\cdot \#}$ represents the sensor where the magnetic field is read. The magnetic gradiometer was interfaced to a Teensy 3.6 microcontroller (PJRC, USA) through the I2C protocol and sampled at a rate of 100 Hz.

## Supplementary Materials

### The PDF file includes:

Note S1 Figs. S1 to S11 Tables S1 to S6 Legends for movies S1 to S6
