# Supplementary material for: Combining tethered and untethered magnetic robots via a magnetically triggerable latch for target payload delivery and retrieval
Source: Sci Adv. 2026 Jan 1;12(1):eadu6025. doi: 10.1126/sciadv.adu6025 (PMC12757063; doi:10.1126/sciadv.adu6025)
Supplement: Supplementary file 1 — Note S1 Figs. S1 to S11 Tables S1 to S6 Legends for movies S1 to S6 [file sciadv.adu6025_sm.pdf]

Science Advances
AAAS

Supplementary Materials for

Combining tethered and untethered magnetic robots via a magnetically triggerable latch for target payload delivery and retrieval

Michael Brockdorff *et al.*

Corresponding author: Michael Brockdorff, mbrock97@gmail.com; Pietro Valdastri, p.valdastri@leeds.ac.uk

Sci. Adv. **12**, eadu6025 (2026)
DOI: 10.1126/sciadv.adu6025

The PDF file includes:

Note S1
Figs. S1 to S11
Tables S1 to S6
Legends for movies S1 to S6

Other Supplementary Material for this manuscript includes the following:

Movies S1 to S6

Note S1. Magnetic latch mechanical durability tests

To confirm that the bonding provided by the flexible epoxy may withstand the forces and torques imparted by the IPM and mUMR during release and (shown in Tables S2 and S4), a mechanical durability test was performed. The testing rig shown in Fig. S10A was set up. This consisted of a fixed clamp, along with a six-axis load cell (Nano 17, ATI Industrial Automation, USA) attached to a motorized linear stage (NRT150/M, Thorlabs, Inc., U.S.A.). The distal end of a 5 cm long, 1.5 mm diameter mSCR was attached to the load cell while the proximal end of the mSCR (containing the magnetic latch) was attached to the fixed clamp. The linear stage was moved so that the load cell moved away from the fixed clamp at a speed of 5 mm/s. The stage was retracted until the mSCR's material broke apart, or the epoxy attaching the magnetic latch to the mSCR failed, whichever came first, as seen in movie S6. The force at which this happened was recorded. This test was repeated six times, three times for magnetic latches made using a flexible epoxy adhesive (IRS 2126, Intertronics, United Kingdom) and three times for magnetic latches made using PVA glue (GL600R Red Label, Brian Clegg, United Kingdom).

The forces required to rupture the mSCRs are shown in Fig. S10C. For the mSCRs with magnetic latches attached using PVA glue, the magnetic latch detached from the mSCR at a median force of 0.45 N. For the mSCRs made using the flexible epoxy the mSCR material broke before the bonding between the mSCR and magnetic latch failed. This happened at an average force of 2.1 N. This justifies the use of the flexible epoxy as a bonding agent as it can more than withstand the 0.32 N that the magnetic latch experiences when releasing and reattaching mUMRs.

The durability of the chosen epoxy was further assessed by conducting the same mechanical durability test after subjecting the magnetic latch to 50 release/re-attachment cycles. The necessary magnetic fields were generated using the tri-axial Helmholtz coil shown in Fig. S1. After these 50 cycles, the mechanical durability test revealed that the mSCR material ruptured before the epoxy of the magnetic latch did.

Supplementary Figures

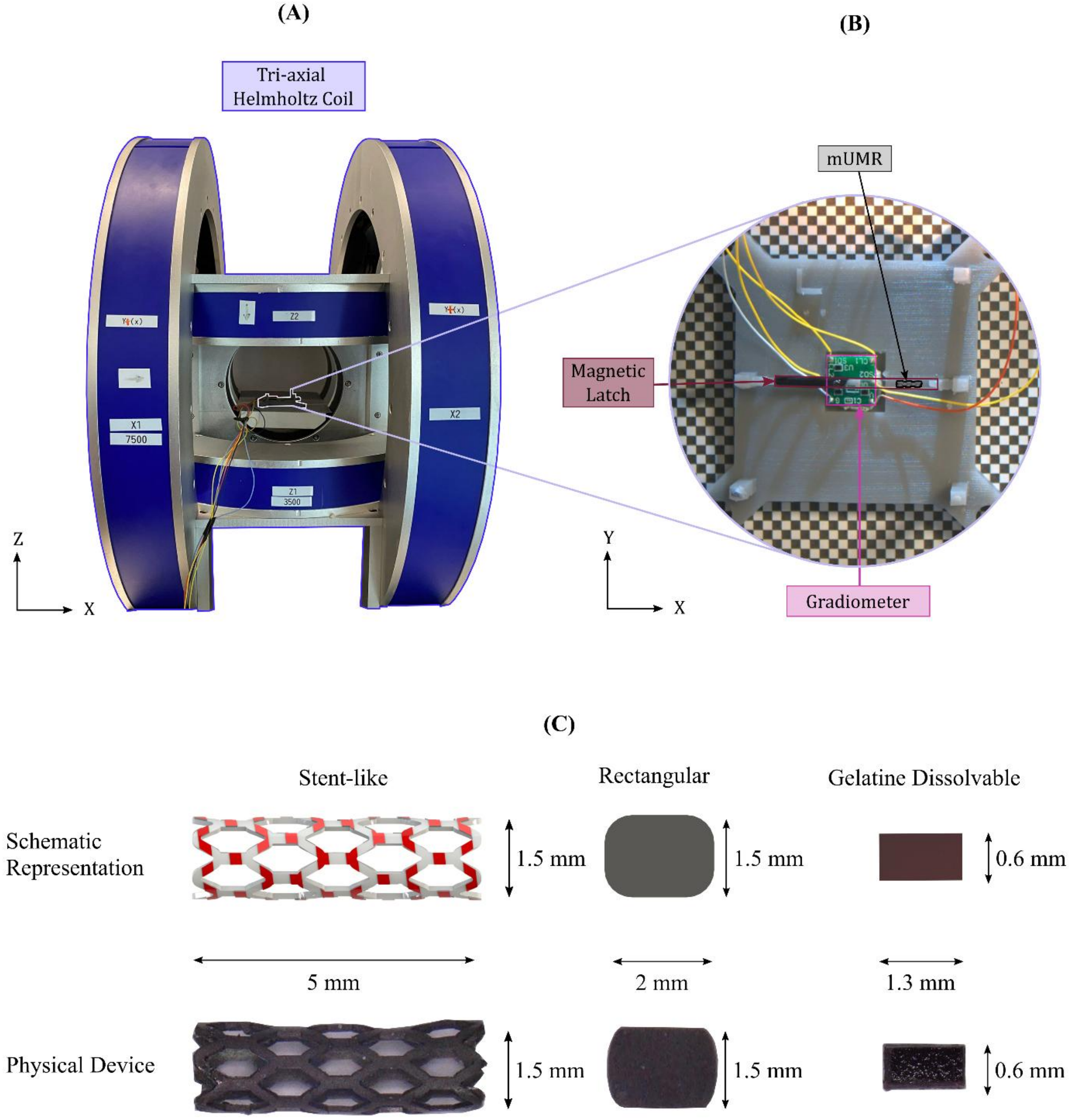

Fig. S1. Magnetic latch characterization setup. (A) The characterization rig was placed within a tri-axial Helmholtz coil system (3DXHC12.5-300, Dexing Magnet Tech. Co.Ltd, China), which provided up to 25 mT in three orthogonal directions. Each individual coil is paired with a bipolar DC power supply (DXKDP, Dexing Magnet Tech. Co. Ltd, China). (**B**) Magnetic latch characterization rig which includes a 3D printed mount to hold the magnetic latch and a magnetic gradiometer. (**C**) Schematic representation of the three mUMRs compared with images of the physical devices, including dimensions.

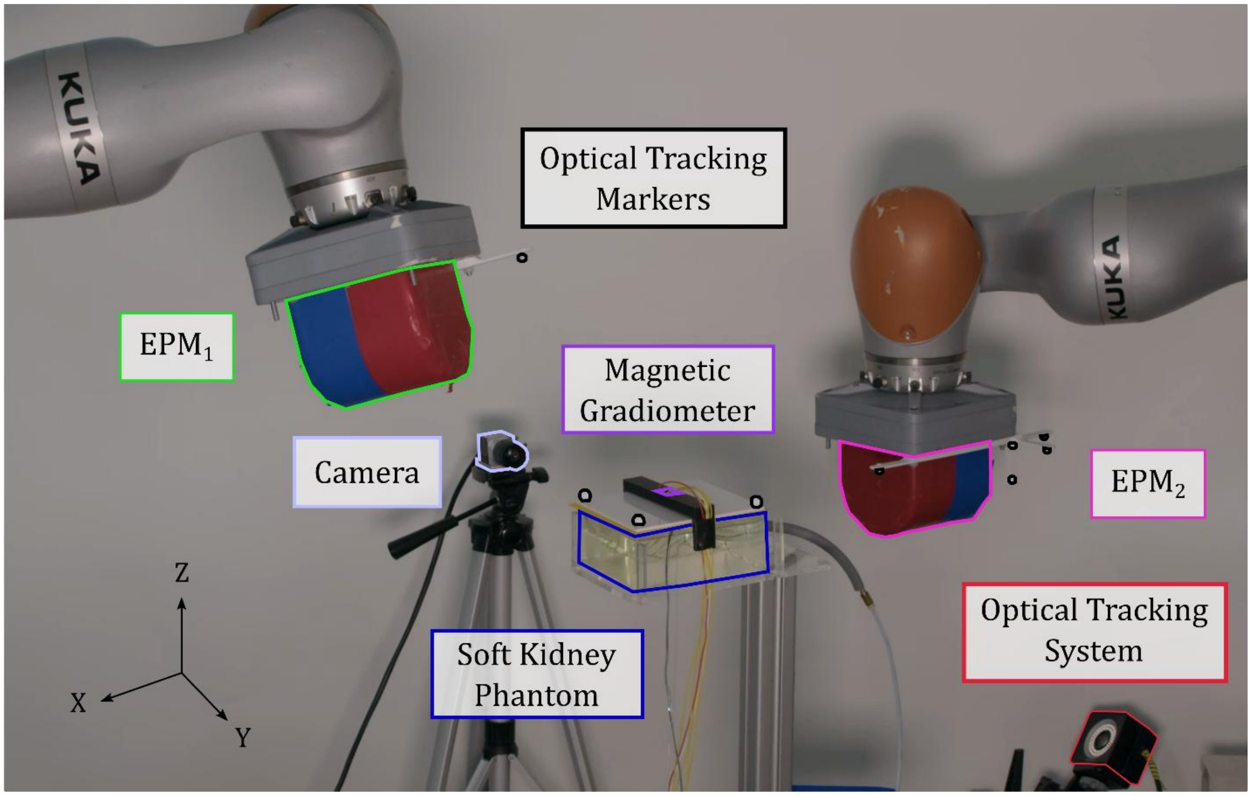

Fig. S2. dEPM platform used to control the navigation of the mSCR and mUMR within a soft kidney phantom.

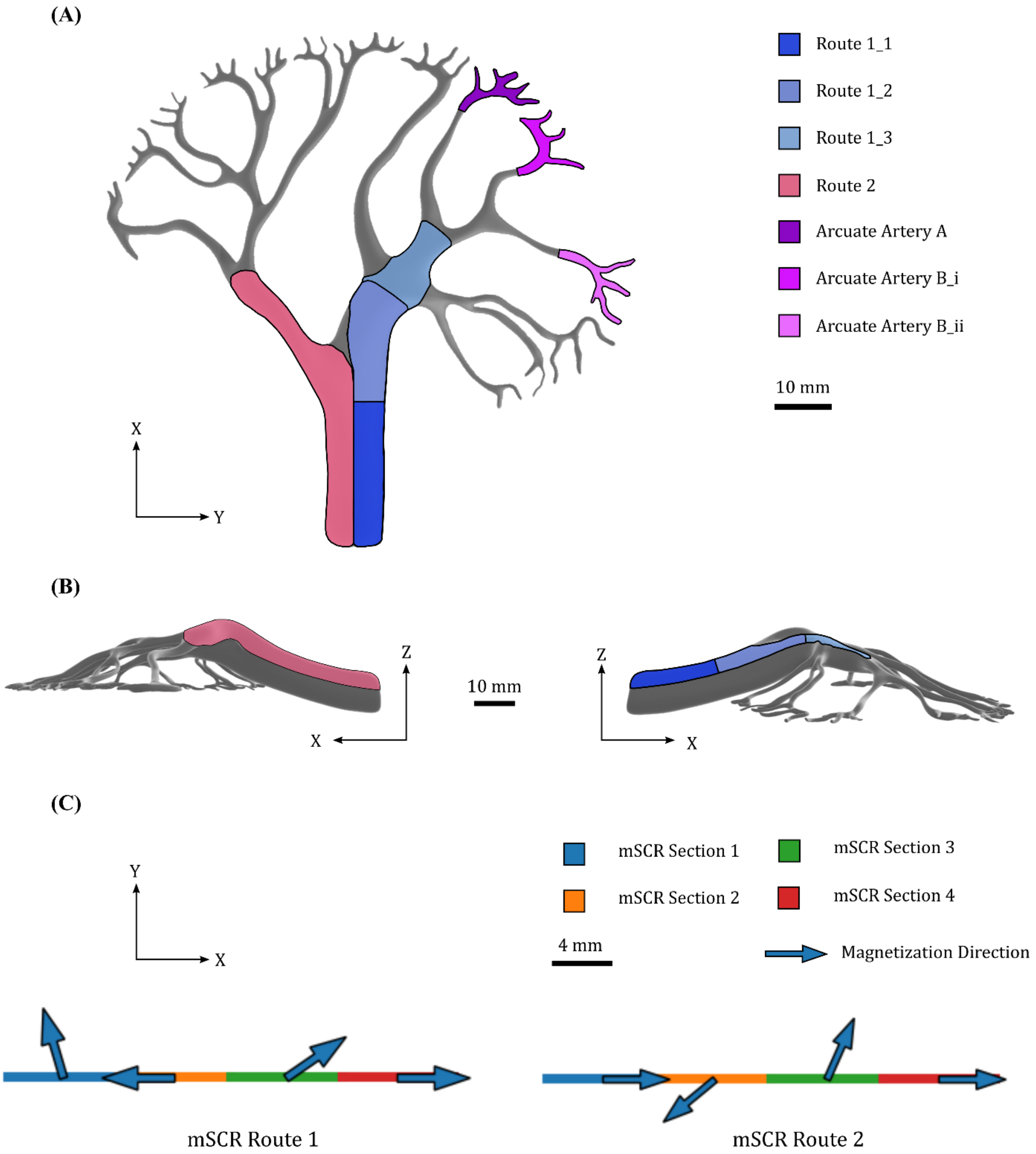

Fig. S3 Path identification and mSCR design for navigation in a soft kidney phantom. (A) 3D representation of the renal artery obtained by segmenting CTA data as seen from above. The routes for each navigation are highlighted in blue and pink for routes 1 and 2 respectively, with the arcuate arteries highlighted in shades of purple. (**B**) Model of the renal artery as seen from the side with intended routes highlighted. (**C**) Magnetic profiles of the two route specific mSCRs.

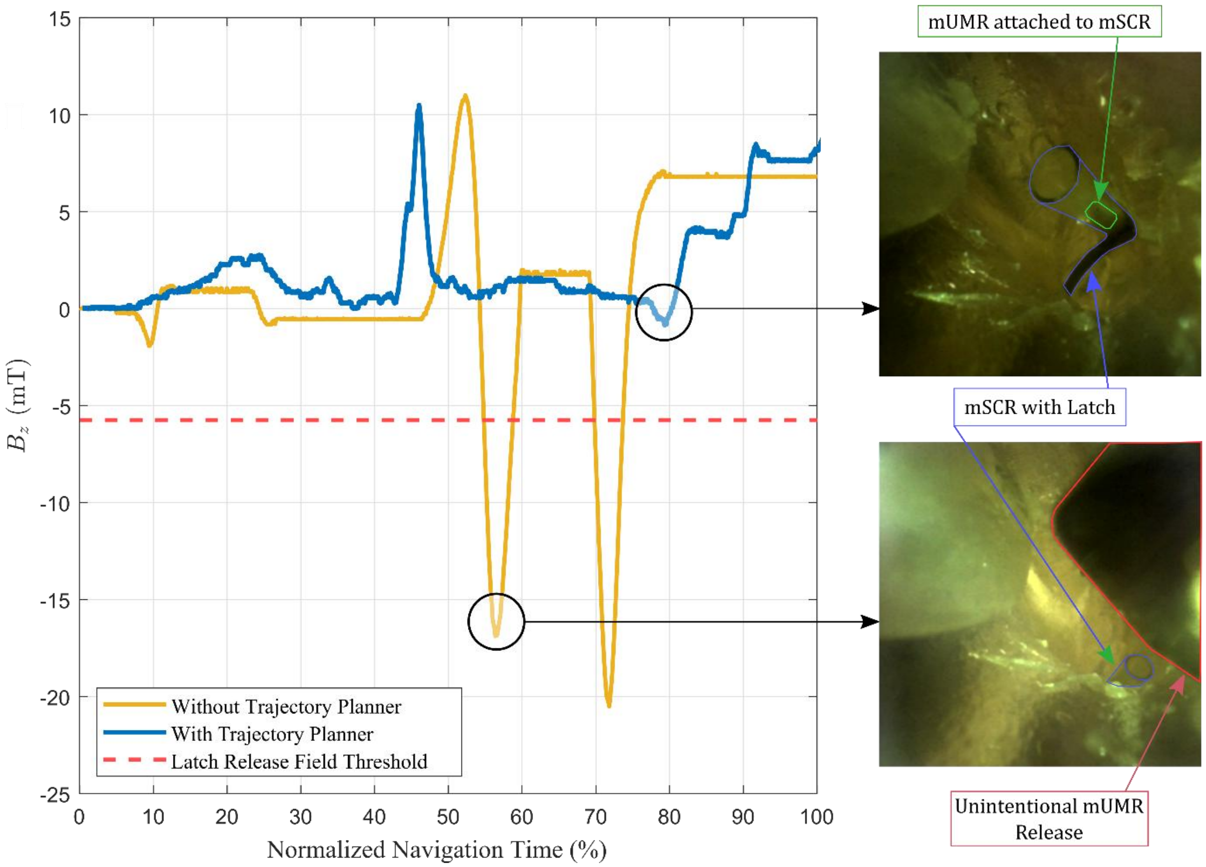

Fig. S4. Navigation in the soft kidney phantom route 1 with and without the hybrid trajectory planner. Without the hybrid trajectory planner, the field along the z-axis (which shares the axis of the release field for the magnetic latch) unintentionally exceeds the latch's release threshold, resulting in an unintended release of the mUMR. However, when using the hybrid trajectory planner, the release field is maintained at 15% of its required value, ensuring the navigation of the mSCR without releasing the mUMR. Once the navigation of the mSCR has been completed the mUMR is released and navigated independently.

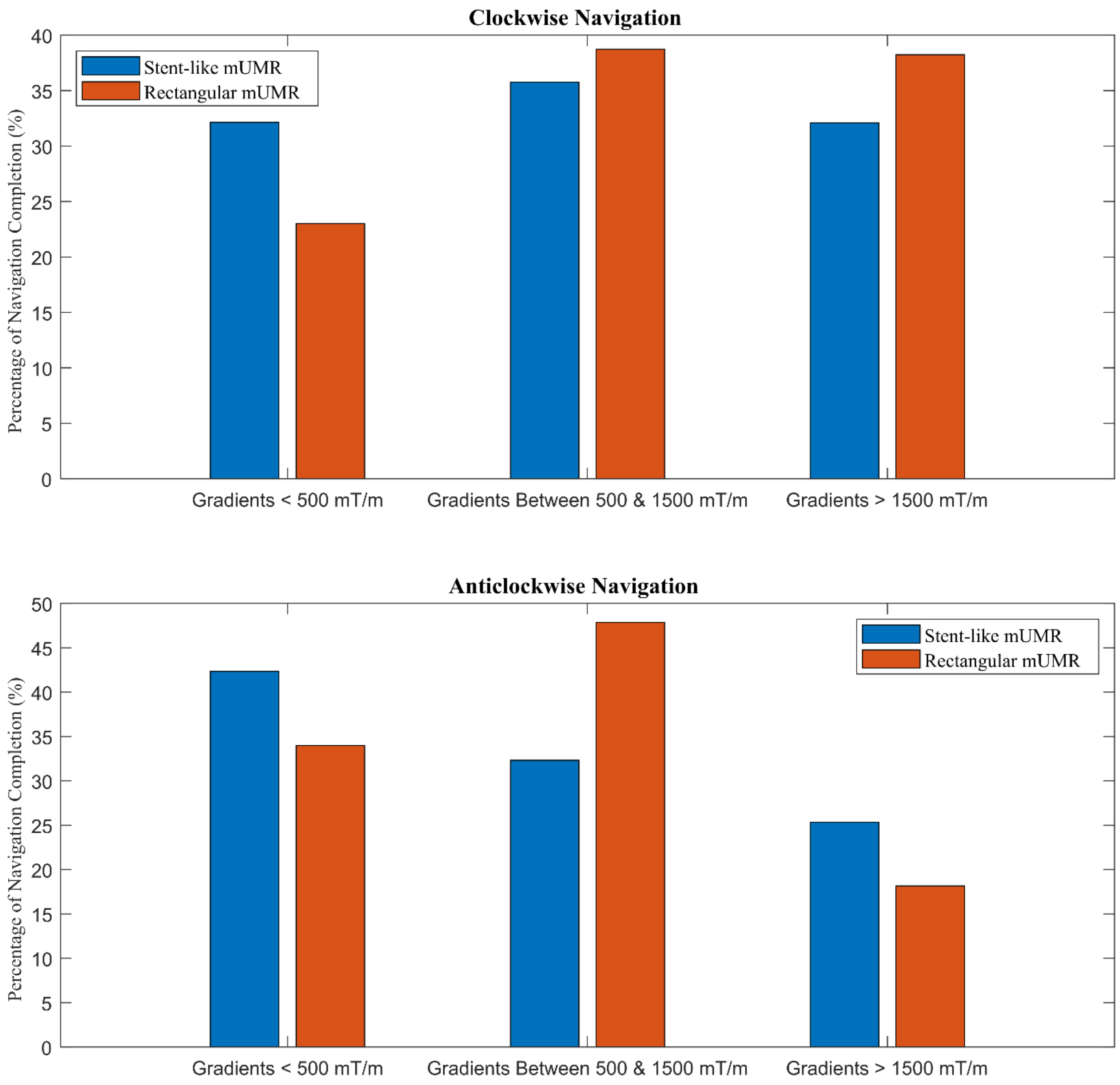

Fig. S5. Average magnetic gradient norm for mUMR navigation against flow for the stent-like and rectangular mUMRs.

(A)

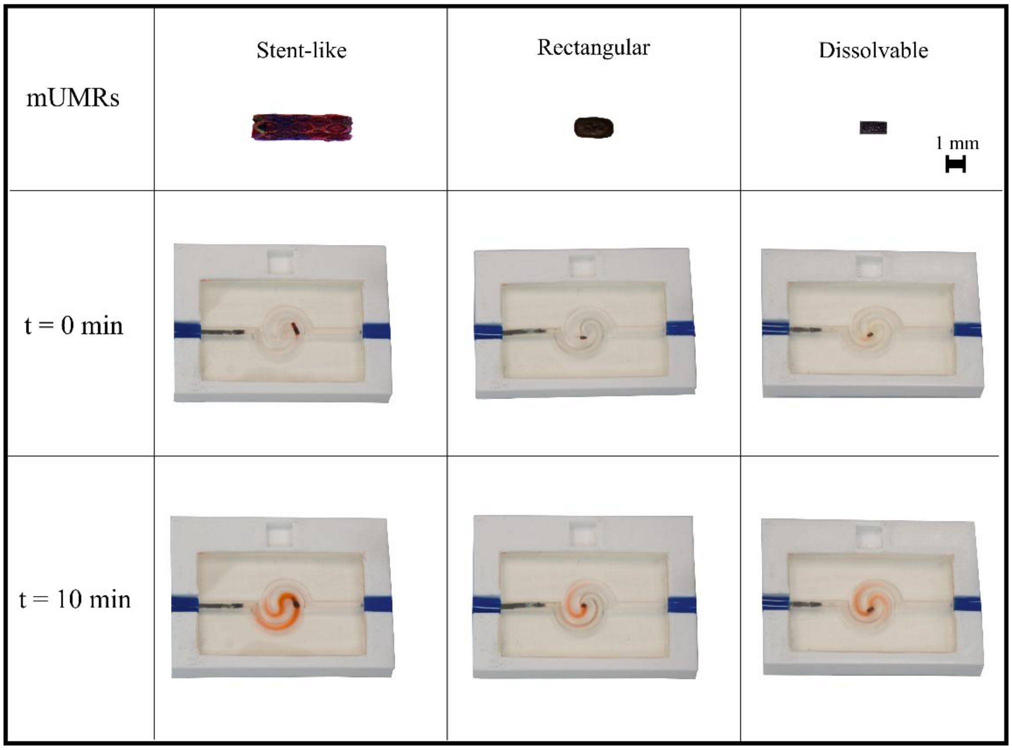

(B)

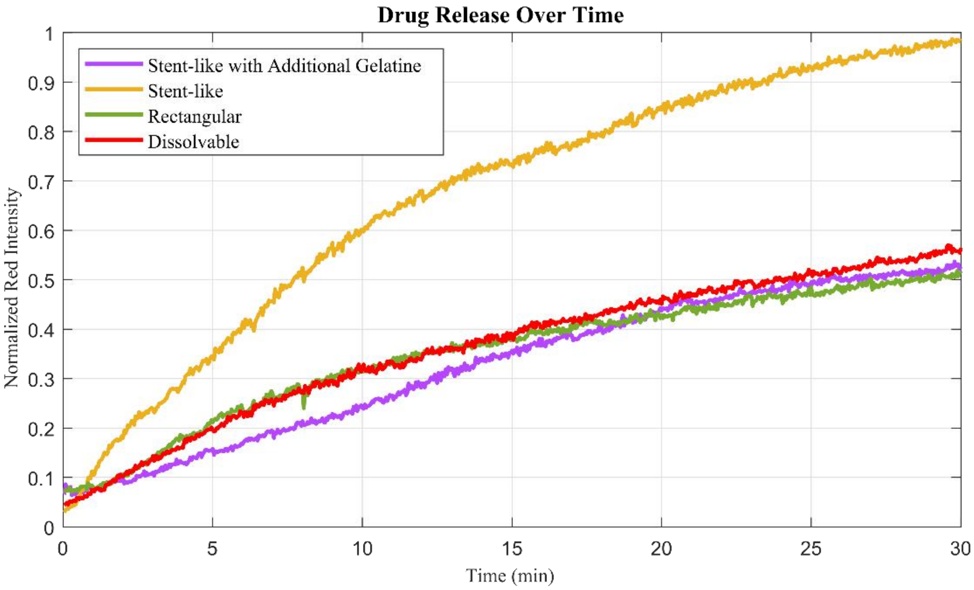

Fig. S6. Mock drug release over time for different mUMRs. (**A**) mUMRs coated with a mixture of red dye and gelatin, used to represent how the mUMRs could be coated with a drug. Snapshots showing the release of the mock drug into the phantom as time passes. (**B**) Graph comparing the release of mock drug between the different payloads over 30 min. The efficiency of drug release was calculated by measuring the ratio of red intensity in the red channel values relative to the number of red pixels in each video frame.

(**A**)

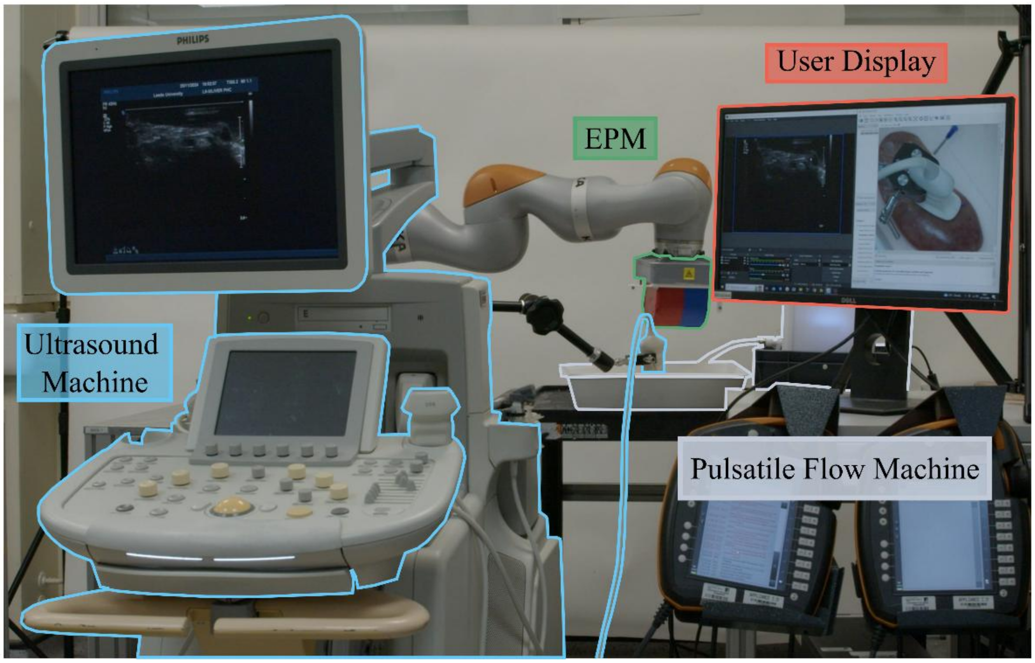

(**B**)

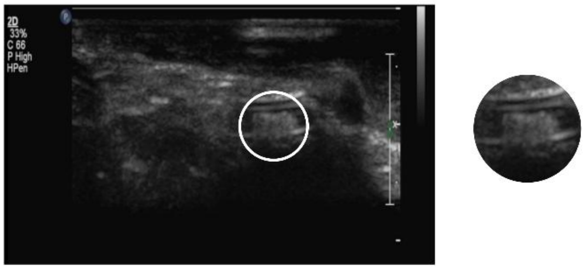

Stent-like mUMR

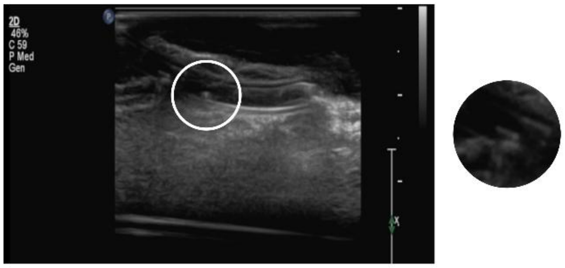

Dissolvable mUMR

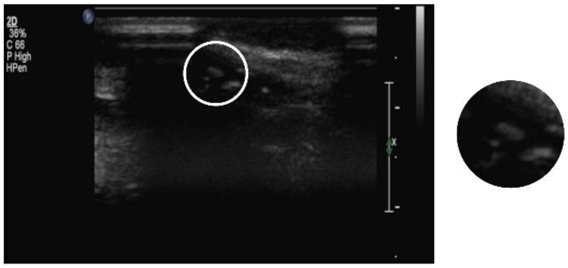

Rectangular mUMR

Fig. S7. Visibility of mUMRs under ultrasound. (A) Experimental setup for the deployment of a stent-like mUMR into a porcine ex vivo kidney under ultrasound guidance. An ultrasound probe was clamped on top of the porcine kidney model, with the ultrasound's output being presented to the surgeon. The surgeon controlled the position of the EPM using a controller. **(B)** Comparison of the visibility of various classes of mUMRs within the vasculature of an *ex vivo* porcine kidney as seen under ultrasound. The stent-like mUMR demonstrates the clearest visibility. The clarity of other mUMRs can be enhanced by incorporating microbubbles into their design.

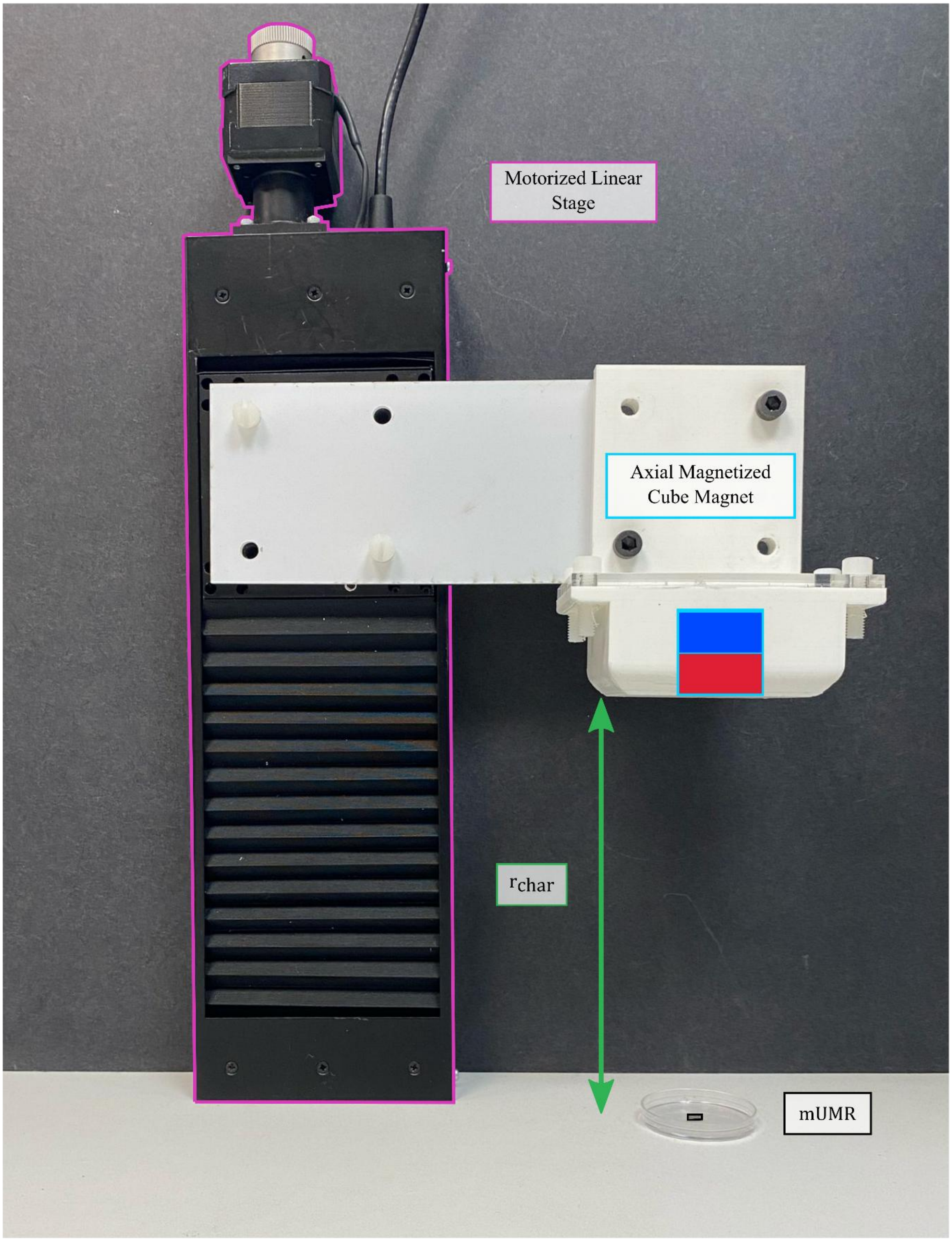

Fig. S8. mUMR magnetic moment characterization setup. A linear motorized stage was used to lower an axial magnetized cube magnet towards the mUMR. Once the magnetic force overcame the gravitational force on the mUMR, the distance r_{char} was measured. This distance along with the mass of the mUMR was used to calculate its magnetic moment.

(A)

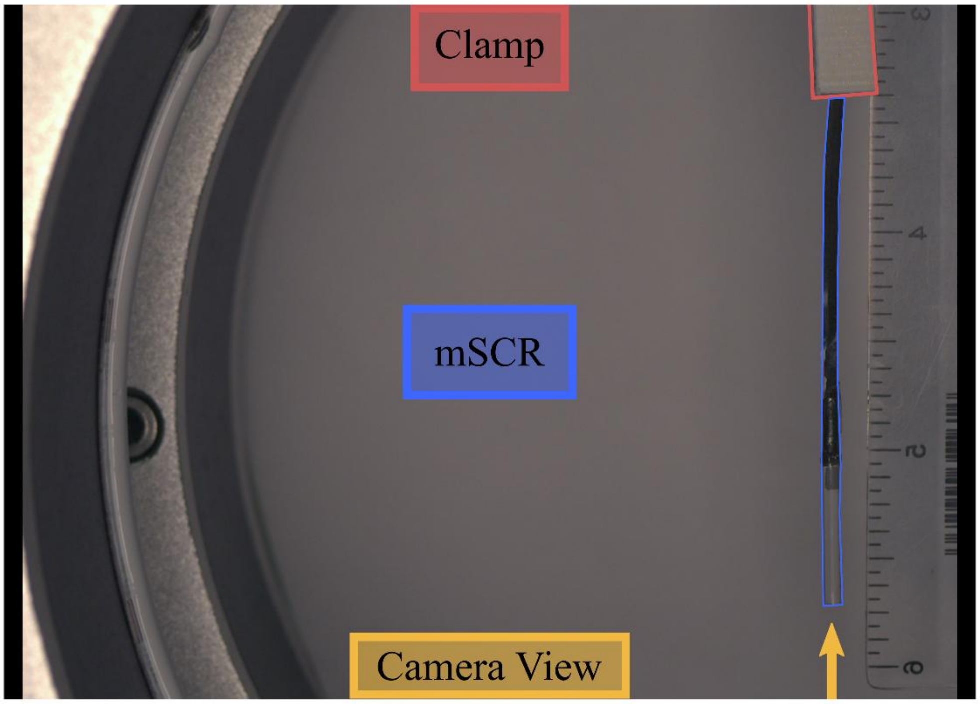

(B)

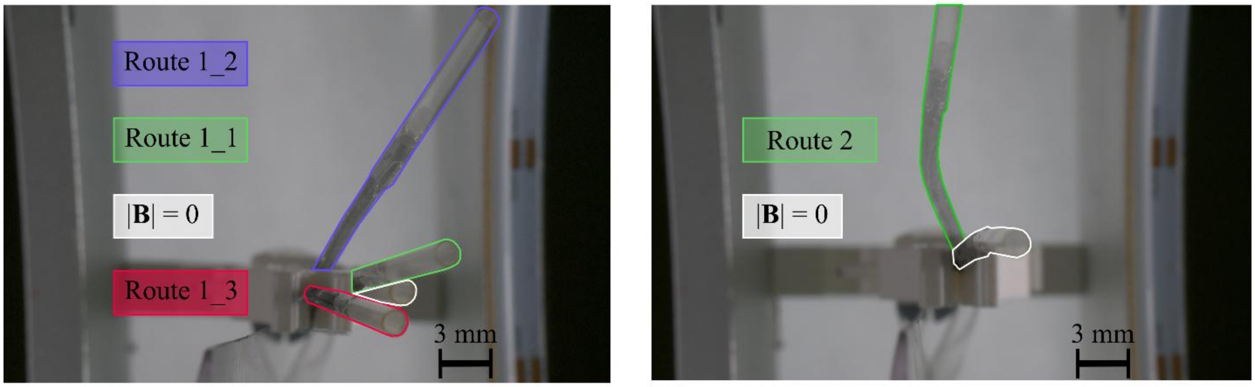

Fig. S9: Out-of-plane deformation characterization. The latch magnet which lies orthogonal to the magnetization plane of the mSCRs, may cause some undesired out-of-plane deformation. (**A**) a tri-axial Helmholtz coil used to generate the required magnetic fields for each section of the navigation path for each mSCR. (**B**) The out-of-plane deformation was measured using a camera placed underneath the mSCRs.

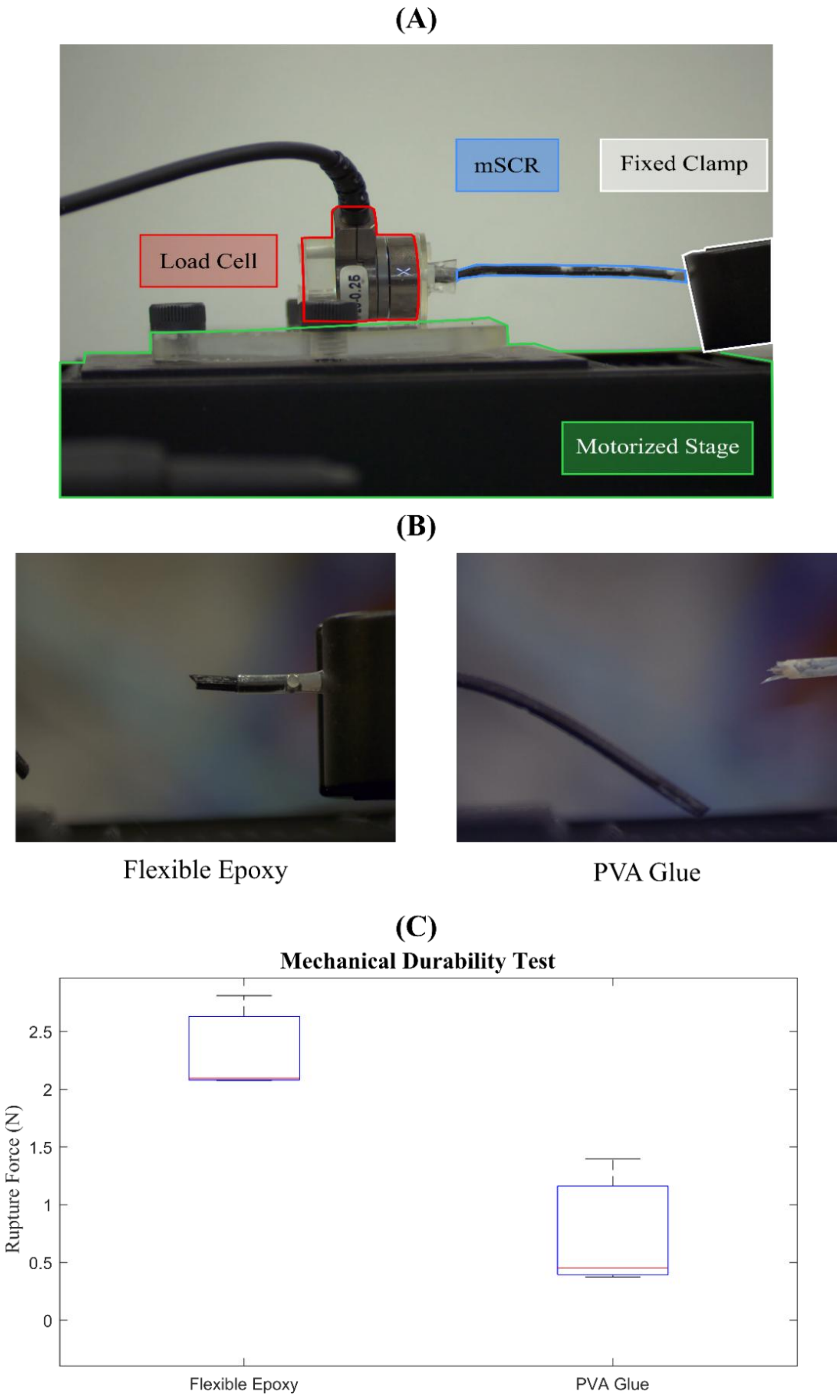

Fig. S10: Mechanical durability test. (A) Setup to test the mechanical durability of different adhesives used when attaching the magnetic latch to the mSCR. (**B**) Different points of failures where observed for the different adhesives. (**C**) Qualitative comparison of the different forces required to rupture the two types of bonding agents.

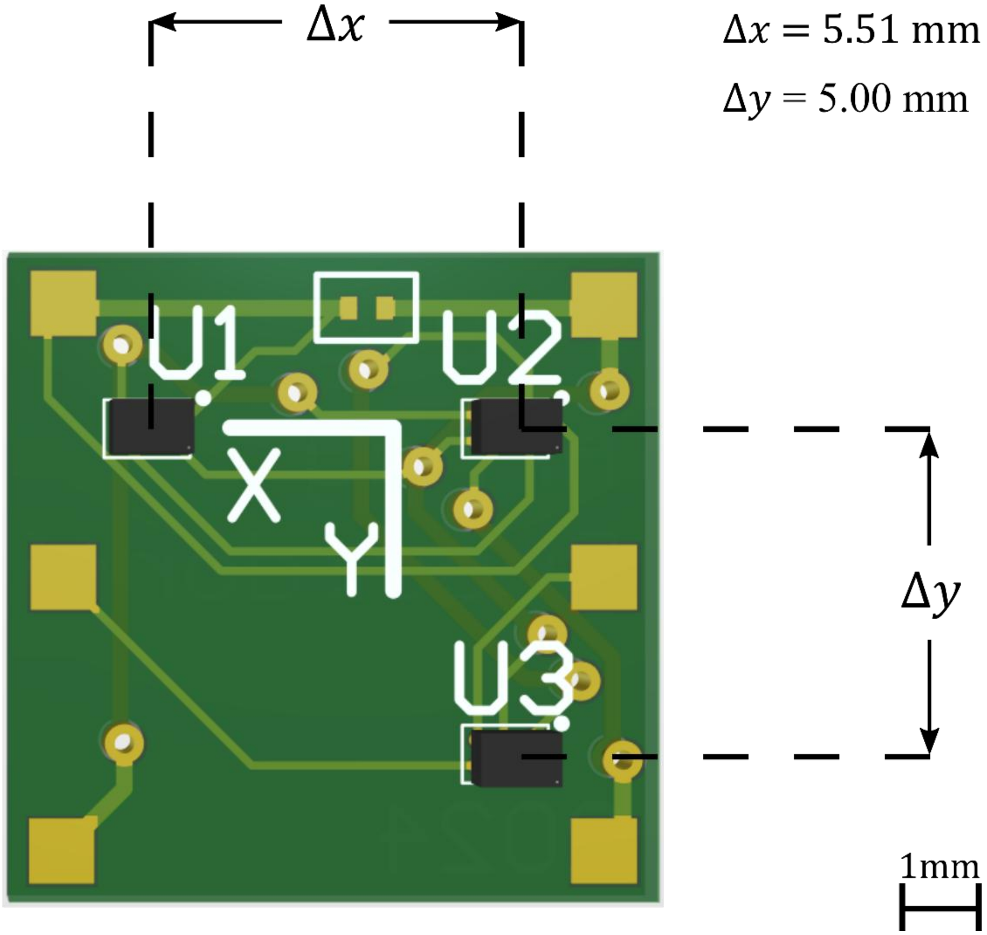

Fig. S11. Magnetic Gradiometer. Consists of three tri-axial magnetic field sensors used to approximate the magnetic field and gradient at the position of the sensor U2 using finite differences.

Supplementary Tables

Table S1. Comparison of magnetic field required to release each type of mUMR from the magnetic latch with varying separation layer thicknesses.

**Name of mUMR**	**Magnetic Moment (Am^2)**	**Release Field (mT)**		
		Separation Layer 2.5 mm	Separation Layer 4 mm	Separation Layer 7.5 mm
Rectangular 1:0.75	3.95×10^{-5}	4.86 ± 0.36	1.60 ± 0.21	1.17 ± 0.37
Rectangular 1:1	5.57×10^{-5}	5.35 ± 0.45	1.49 ± 0.12	0.98 ± 1.00
Rectangular 1:1.5	8.58×10^{-5}	5.50 ± 0.44	1.82 ± 0.15	1.65 ± 0.28
Dissolvable	4.41×10^{-5}	10.04 ± 0.85	2.91 ± 0.16	0.94 ± 0.19
Stent-like	1.27×10^{-4}	2.50 ± 0.67	2.01 ± 0.94	

Table S2: Estimated latching force for each class of mUMR at different separation layer thicknesses. The latching force was calculated using the approximation shown in (4).

**Name of mUMR**	**Magnetic Moment (Am^2)**	**Estimated Latching Force $\|\|f_{latch}\|\|$ (mN)**		
		Separation Layer 2.5 mm	Separation Layer 4 mm	Separation Layer 7.5 mm
Rectangular 1:0.75	3.95×10^{-5}	0.149	0.036	0.004
Rectangular 1:1	5.57×10^{-5}	0.210	0.051	0.006
Rectangular 1:1.5	8.58×10^{-5}	0.324	0.078	0.009
Dissolvable	4.41×10^{-5}	0.254	0.053	0.006
Stent-like	1.27×10^{-4}	0.115	0.040	

Table S3: Estimated torque needed to rotate each mUMR and hence, release it from the magnetic latch for different separation layer thicknesses. The torque was calculated using the dipole approximation for magnetic torque.

**Name of mUMR**	**Estimated Release Torque** $\|\|\boldsymbol{\tau}\|\|$ (mN.m^{-1})		
	Separation Layer 2.5 mm	Separation Layer 4 mm	Separation Layer 7.5 mm
Rectangular 1:0.75	0.1920	0.0632	0.0462
Rectangular 1:1	0.2980	0.0830	0.0546
Rectangular 1:1.5	0.4867	0.1611	0.1460
Dissolvable	0.4428	0.1283	0.0415
Stent-like	0.3175	0.2553	

Table S4: Characterization of the maximum out-of-plane deformation produced by the tip magnet, which actuates the latching system.

**Navigation Route**	Route 1_1	Route 1_2	Route 1_3	Route 2
**Out-of-plane deformation (mm)**	2.55	4.85	0.65	3.96

Table S5. The magnetic field required to navigate each mSCR through their respective routes.

Navigation Route	Magnetic Field (mT)		
	Bx	By	Bz
Route 1_1	0.5	-3.0	1.0
Route 1_2	15.0	0	1.75
Route 1_3	3.5	3.0	7.25
Route 2	12.5	-0.5	-5

Table S6. Average navigation times for the stent-like and rectangular mUMRs in the vascular phantom. The mUMRs were navigated into two directions, clockwise and counterclockwise. Re-attachment to the latch was always done against flow.

	**Clockwise**		**Counterclockwise**	
	Stent-like	Rectangular	Stent-like	Rectangular
**Release (min)**	2.88 ± 0.98	2.03 ± 0.75	1.41 ± 1.34	0.75 ± 0.92
**Navigation (min)**	11.84 ± 6.30	5.37 ± 5.65	5.60 ± 6.06	4.42 ± 2.37
**Re-attachment (min)**	8.26 ± 1.11	10.39 ± 10.07	7.71 ± 9.30	7.44 ± 2.31

Supplementary Movies

Movie S1. Magnetic latch characterization and release of different mUMRs.
This video demonstrates the characterization setup for the magnetic latch, showing the release of stent-like, rectangular, and dissolvable mUMRs.

Movie S2. mSCR navigation in a soft kidney phantom.
This video demonstrates the navigation of two mSCRs along different routes in a soft kidney phantom, using the hybrid trajectory planner. Without the planner, the mUMR is prematurely released from the magnetic latch before the mSCR reaches its final destination.

Movie S3. Integrated mSCR navigation with mUMR deployment and retrieval.
This video shows the navigation of an mSCR with an integrated magnetic latch with a clear, 3D printed, kidney phantom. Once successfully progressing through route 1 of the phantom, the mUMR is released from the magnetic latch and independently navigated to the arcuate arteries. Once navigation is complete the mUMR is navigated back to the mSCR were it reattaches to the magnetic latch and is removed from the anatomy.

Movie S4. Influence of blood flow on mUMR navigation and retrieval.
This video demonstrates the navigation of rectangular and stent-like mUMRs both with and against flow at a rate of 1.3 L/min. It also shows how mUMRs can reattach to the magnetic latch against the flow. Additionally, the effect of insufficient magnetic coupling between the mUMR and EPM is demonstrated.

Movie S5. Delivery and retrieval of a stent-like mUMR under ultrasound guidance.
This video demonstrates the ultrasound-guided release and reattachment of a stent-like mUMR in a porcine kidney model. The impact of insufficient magnetic coupling between the mUMR and EPM is also shown.

Movie S6. Magnetic latch mechanical durability tests.
This video demonstrates the durability of the magnetic latch, showcasing the difference when using a flexible epoxy compared to PVA glue as a magnetic latch bonding agent.
